# GAP43 Located on Corticostriatal Terminals Restrains Novelty-Induced Hyperactivity in Mice

**DOI:** 10.1523/JNEUROSCI.0701-24.2024

**Published:** 2024-08-21

**Authors:** Irene B. Maroto, Carlos Costas-Insua, Carlos Montero-Fernández, Alba Hermoso-López, Margaux Lebouc, Raquel Bajo-Grañeras, Alicia Álvaro-Blázquez, Cristina Blázquez, Astrid Cannich, Giovanni Marsicano, Ricardo Martín, Jérôme Baufreton, Ignacio Rodríguez-Crespo, Luigi Bellocchio, Manuel Guzmán

**Affiliations:** ^1^Department of Biochemistry and Molecular Biology, Schools of Biology and Chemistry, Instituto Universitario de Investigación Neuroquímica (IUIN), Complutense University, Madrid ES-28040, Spain; ^2^Centro de Investigación Biomédica en Red de Enfermedades Neurodegenerativas (CIBERNED), Instituto de Salud Carlos III, Madrid ES-28029, Spain; ^3^Instituto Ramón y Cajal de Investigación Sanitaria (IRYCIS), Madrid ES-28034, Spain; ^4^University of Bordeaux, CNRS, IMN, UMR 5293, Bordeaux F-33000, France; ^5^Department of Physiology, School of Medicine, Instituto Universitario de Investigación Neuroquímica (IUIN), Complutense University, Madrid ES-28040, Spain; ^6^University of Bordeaux, INSERM, Neurocentre Magendie, U1215, Bordeaux F-33000, France

**Keywords:** cannabinoid, corticostriatal circuitry, GAP43, glutamatergic transmission, long-term depression, motor activity

## Abstract

Growth-associated protein of 43 kDa (GAP43) is a key cytoskeleton-associated component of the presynaptic terminal that facilitates neuroplasticity. Downregulation of GAP43 expression has been associated to various psychiatric conditions in humans and evokes hippocampus-dependent memory impairments in mice. Despite the extensive studies conducted on hippocampal GAP43 in past decades, however, very little is known about its roles in modulating the excitatory versus inhibitory balance in other brain regions. We recently generated conditional knock-out mice in which the *Gap43* gene was selectively inactivated in either telencephalic glutamatergic neurons (*Gap43^fl/fl^*;*Nex1^Cre^* mice, hereafter Glu-GAP43^−/−^ mice) or forebrain GABAergic neurons (*Gap43^fl/fl^*;*Dlx5/6^Cre^* mice, hereafter GABA-GAP43^−/−^ mice). Here, we show that Glu-GAP43^−/−^ but not GABA-GAP43^−/−^ mice of either sex show a striking hyperactive phenotype when exposed to a novel environment. This behavioral alteration of Glu-GAP43^−/−^ mice was linked to a selective activation of dorsal-striatum neurons, as well as to an enhanced corticostriatal glutamatergic transmission and an abrogation of corticostriatal endocannabinoid-mediated long-term depression. In line with these observations, GAP43 was abundantly expressed in corticostriatal glutamatergic terminals of wild-type mice. The novelty-induced hyperactive phenotype of Glu-GAP43^−/−^ mice was abrogated by chemogenetically inhibiting corticostriatal afferences with a G_i_-coupled “designer receptor exclusively activated by designer drugs” (DREADDs), thus further supporting that novelty-induced activity is controlled by GAP43 at corticostriatal excitatory projections. Taken together, these findings show an unprecedented regulatory role of GAP43 in the corticostriatal circuitry and provide a new mouse model with a delimited neuronal-circuit alteration for studying novelty-induced hyperactivity, a phenotypic shortfall that occurs in diverse psychiatric diseases.

## Significance Statement

Psychiatric alterations such as attention deficit/hyperactivity disorder, schizophrenia, and bipolar disorder pose a significant health and socioeconomic burden to our society. Animal models that recapitulate precise phenotypic traits of those diseases are therefore warranted for developing new therapeutic interventions. Here, we found that mice lacking the protein GAP43 selectively in telencephalic glutamatergic neurons show a robust novelty-induced hyperactive phenotype, a behavioral deficit often associated to psychiatric diseases. These mice exhibit profound alterations in corticostriatal excitatory plasticity and a selective overactivation of dorsal-striatum neurons in response to a novel environment. Our findings thus unveil an important role of GAP43 in corticostriatal function and provide a new animal model with a delimited neuronal-circuit alteration for studying novelty-induced hyperactivity in psychiatric disorders.

## Introduction

Growth-associated protein of 43 kDa (GAP43; aka neuromodulin) is a long-known important mediator of axonal neuroplastic and regenerative responses. GAP43 is a cytoskeleton-associated, synaptic vesicle-bound protein that shows a ubiquitous expression throughout the mouse central nervous system, mainly at presynaptic terminals (Holahan, 2017). Upon its activation by protein kinase C-mediated phosphorylation, GAP43 can interact with many proteins to facilitate presynaptic rearrangements (He et al., 1997; Neve et al., 1998). GAP43 is a key factor in neurite outgrowth during brain development and orchestrates the amplification of pathfinding signals from numerous adhesion molecules and growth factors in the axonal growth cone (Korshunova and Mosevitsky, 2010). Thus, interfering with GAP43 function inhibits developmental neurite extension in mice (Benowitz and Routtenberg, 1997) and, accordingly, knocking out GAP43 in the germline results in lethality at embryonic or early postnatal stages in mice (Strittmatter et al., 1995).

GAP43 also facilitates neural plasticity and axonal regeneration in the mouse and rat adult brain. At this life stage, GAP43 expression remains elevated in areas that undergo recurrent input-dependent plasticity such as the hippocampus, neocortex, olfactory bulb, cerebellum, amygdala, and basal ganglia (Benowitz et al., 1988; Kruger et al., 1993). However, functional studies on GAP43-mediated plasticity in the adult brain have been conducted almost exclusively in the hippocampal formation. Thus, induction of long-term potentiation increases GAP43 levels (Meberg et al., 1995; Namgung et al., 1997) and phosphorylation (Lovinger et al., 1985; Routtenberg et al., 1985; Gianotti et al., 1992; Ramakers et al., 1999) at various hippocampal synapses. In addition, expression of a phosphomimic, constitutively active form of GAP43 enhances hippocampal long and short-term plasticity (Routtenberg et al., 2000; Hulo et al., 2002). Likewise, GAP43 overexpression boosts memory-associated tasks, while either expression of a nonphosphorylatable, constitutively inactive form of GAP43, or downregulation of GAP43 expression induces deficits in hippocampal-dependent memory and learning (Routtenberg et al., 2000; Rekart et al., 2005). Dysregulation of GAP43 function in the rat hippocampus has been associated as well to epileptic phenotypes and an aberrant sprouting of mossy fibers (McNamara and Routtenberg, 1995). Likewise, a prominent modulatory role of GAP43 on cannabinoid CB_1_ receptor (CB_1_R)-mediated plasticity at terminals of mossy cells in the mouse dentate gyrus, with a closely associated effect on seizure susceptibility, has been recently reported (Maroto et al., 2023).

GAP43 haploinsufficiency in mice also causes connectivity-associated alterations such as an aberrant functional somatotopy (Dubroff et al., 2006), multiple sensorimotor deficits, anxiety, and decreased sociability (Zaccaria et al., 2010). Of note, altered GAP43 levels have been found in mouse models and patients with various neuropsychiatric conditions, including schizophrenia (Weickert, 2001), bipolar disorder (Tian et al., 2007), attention deficit/hyperactivity disorder (ADHD; Almeida et al., 2023), and cortical dysplasia-associated seizures (Nemes et al., 2017). These neuropsychiatric diseases are highly complex, involving multiple brain areas and heterogeneous phenotypes that are frequently associated with a misadjusted excitatory power (Gao and Penzes, 2015). However, despite the high levels of GAP43 expression found in many brain areas, very little is known about the functions of GAP43 outside the hippocampal formation and the potential contribution of GAP43 to the excitatory versus inhibitory imbalance that underlies the aforementioned neuropsychiatric traits. We have recently generated conditional knock-out mice in which the GAP43-encoding gene was selectively inactivated in either telencephalic glutamatergic neurons (*Gap43^fl/fl^*;*Nex1^Cre^* mice, hereafter referred to as Glu-GAP43^−/−^) or forebrain GABAergic neurons (*Gap43^fl/fl^*;*Dlx5/6^Cre^* mice, hereafter referred to as GABA-GAP43^−/−^ mice; Maroto et al., 2023). Using these mouse lines, here we report that the pool of GAP43 molecules selectively located on corticostriatal glutamatergic terminals is necessary to maintain a proper basal transmission and plasticity of those synapses and to restrain novelty-induced hyperactivity, thus unveiling an unprecedented role of GAP43 in the corticostriatal circuitry.

## Materials and Methods

### Animals

Experimental procedures were performed in accordance with the guidelines and approval of the Animal Welfare Committees of Universidad Complutense de Madrid, and Comunidad de Madrid, the directives of the Spanish Government, and the European Commission (protocol code PROEX 209/18, date of approval 25/02/2019). Throughout the study, animals had unrestricted access to food and water. They were housed (typically, 4–5 mice per cage) under controlled temperature (range, 20–22°C), humidity (range, 50–55%), and light/dark cycle (12 h/12 h). Animal housing, handling, and assignment to the different experimental groups was conducted by standard procedures. Adequate measures were taken to minimize pain and discomfort of the animals. Mice harboring a conditional-ready floxed *Gap43* allele in C57BL/6N background were generated in our lab starting from B6Dnk;B6Brd;B6N-Tyrc-Brd *Gap43^tm1a(EUCOMM)Wtsi/WtsiBiat^* mice (EMMA Mouse Repository; MGI ID #5700649). The resulting *Gap43^fl/fl^* mice (hereafter GAP43^fl/fl^ mice) were crossed with *Nex1*-Cre or *Dlx5/6*-Cre-expressing mice, thus yielding the corresponding Glu-GAP43^−/−^ and GABA-GAP43^−/−^ conditional knock-out mouse lines, respectively (Maroto et al., 2023).

### Behavioral tests

Adult (ca. 3-month-old) Glu-GAP43^−/−^ and GABA-GAP43^−/−^ mice, as well as their respective control GAP43^fl/fl^ littermates, of both sexes (at approximately 1:1 ratio within each experimental group, and differentially represented in each figure panel), were used for behavioral tests. Animals were assigned randomly to the different treatment groups, habituated to the experimental room, and handled 1 week before testing. All the behavioral tests were conducted during the early light phase under dim illumination (<50 lux in the center of the corresponding maze) and video recorded for subsequent blind analysis by a different trained observer, using Smart3.0 Software (Panlab).

#### Motor performance tests

Spontaneous locomotor activity was measured in an open-field arena of 70 × 70 cm built in-house with gray plexiglass. Mice were placed in the center of the arena and allowed free exploration for up to 2 h. Total distance traveled, global activity, resting time, maximum speed, and entries in the central part of the arena (25 × 25 cm) were measured. In some experiments, mice were injected intraperitoneally with vehicle [1% (v/v) DMSO in 1:18 (v/v) Tween 80/saline] or Δ^9^-tetrahydrocannabinol (THC; 3 mg/kg body weight; THC Pharm) 30 min prior to the test. To assess motor coordination, we conducted an accelerating RotaRod paradigm consisting of three daily sessions, with a 40 min intertrial interval, for 3 consecutive days. Briefly, the mouse was placed in the rod (Panlab #LE8205) at a constant speed (4 rpm), which was then accelerated (4–40 rpm in 300 s) once the mouse was put in place, and the time that the mouse remained in the rod was measured. Data from trials 4–9 (Days 2 and 3) were pooled and averaged for each animal. To assess muscle strength, we used a hanging wire test. The animal was suspended by its forelimbs on a 45-cm-long metal wire stretched between two posts, which were placed 50 cm above a cage filled with bedding material. The time (in s) until the animal fell was recorded up to 5 min and multiplied by the animal weight (in g) to measure holding impulse.

#### Circadian modulation of locomotor activity

Mice were placed individually in transparent plastic cages (21 × 11 × 17 cm) with a grid floor and food and water dispensers 1 d prior to the test for habituation. Two horizontal lines of infrared captors (two for each line; interline distance, 25 mm; distance between two captors, 12.5 cm) were mounted along each of the longer side walls. The cages were illuminated 12 h per day starting from 7 A.M. The rack was connected to an electronic interface that was communicated to a computer for automatic data storing. Each mouse was introduced into an activity cage, for habituation for 2 h, and left undisturbed from 6 P.M. for the following 72 h. The mean of the 72 h was analyzed in 1 h blocks with the 12 h light/dark phase as a further within-subjects factor. Locomotor activity was evaluated based on the number of breaks of the infrared captors and expressed in arbitrary units.

#### Spontaneous Y-maze alternation test

Spontaneous alternation was assessed in a gray, plastic (41 × 8 cm) three-arm Y-maze with three opaque 15 cm high and 0.5 cm thick arms orientated at 120° angles from one another, located in a room containing a variety of extra-maze cues. Mice were introduced at the bottom of one arm and allowed to freely explore the maze for 5 min. The starting arm was randomized between tests. Total arm entries were measured. Spontaneous alternation of arm entries represented the proportion of arm choices differing from the previous two versus all consecutive three-arm alternations performed by the animal and was expressed as a percentage.

#### Sociability tests

To evaluate social behavior, mice were isolated in individual cages for 3 d. The test day, a single mouse was introduced in an arena (60-cm-long, 40-cm-wide, 40-cm-high walls) divided into three compartments (20 cm long each) separated by two walls (15 cm long) with a connector corridor (10 cm wide) and containing two cylindrical cages (15 cm high, 8.5 cm diameter) in the lateral compartments, and it was allowed to freely explore the arena and the empty cages (Empty 1 or Empty 2) for 5 min. Mice did not show any preference for any of the empty cages. One hour later, the mouse was re-exposed to this environment, but one of the cages contained one unfamiliar mouse (Subject 1), paired in sex and age, to evaluate sociability expressed as the preference for a mate. Mouse behavior was recorded for 5 min. One hour later, the mouse was re-exposed to the previous environment with Subject 1, but this time the previously empty cage contained a new unfamiliar mouse (Subject 2) to evaluate preference for social novelty. Mouse behavior was again recorded for 5 min. For both trials, the time spent sniffing within each cage was annotated manually by a blinded experimenter using a chronometer. The position of the cages containing the mice was randomized.

### Western blotting

Mouse brains were dissected, and the whole cortex, dorsal striatum, and hippocampal formation were collected and snap frozen. Then, samples were prepared on ice-cold lysis buffer (50 mM Tris-HCl, 0.1% Triton X-100, 1 mM EDTA, 1 mM EGTA, 50 mM NaF, 10 mM Na-β-glycerophosphate, 5 mM Na-pyrophosphate, and 1 mM Na-orthovanadate, pH 7.5) supplemented with a protease-inhibitor cocktail (Roche), 0.1 mM PMSF, 0.1% β-mercaptoethanol, and 1 µM microcystin. Tissue lysates were clarified by centrifugation at 12,000 × *g* for 15 min (4°C), and total protein concentration was determined by the Bradford assay. Then, 10–20 µg of total protein aliquots were mixed with 5× Laemmli sample buffer and boiled at 95°C for 5 min (except for vGluT1 and vGAT detection, according to antibody manufacturer's recommendations). Equal amounts of protein samples were resolved on 12% SDS–PAGE and transferred to polyvinylidene difluoride (PVDF) membranes (Bio-Rad). After incubation for at least 1 h in blocking buffer containing 5% w/v bovine serum albumin (BSA) in Tris-buffered saline-Tween 20 (TBS-T), membranes were blotted overnight at 4°C with the following primary antibodies and dilutions: mouse anti-*pan*-GAP43 (1:1,000, SCBT #33705), rabbit anti-vGluT1 (1:1,000, Synaptic Systems, #135303), rabbit anti-vGAT (1:1,000, Synaptic Systems, #131003), mouse anti-PSD95 (1:1,000, Abcam, #ab2723), rabbit anti-GAPDH (1:3,000, CST #2118), mouse anti-α-tubulin (1:10,000, Sigma-Aldrich, #T9026). All antibodies were prepared in TBS-Tween 20 (0.1%) with 5% BSA w/v. PVDF membranes were then rinsed three times with TBS-T and incubated with the corresponding mouse or rabbit secondary antibodies coupled to horseradish peroxidase (mouse IgG HRP-linked antibody, 1:5,000, Sigma-Aldrich, #NA-931-1; rabbit IgG HRP-linked antibody, 1:5,000, Sigma-Aldrich, #NA-934V) for 1.5 h at room temperature. After washing three times for 10 min with TBS-T, membranes were developed using an enhanced chemiluminescence kit (Bio-Rad). Densitometric analysis of the relative expression of the protein of interest versus the corresponding loading control was performed with Fiji ImageJ open-source software (NIH). Western blot images were cropped for clarity. Electrophoretic migration of molecular weight markers is depicted on the left side of each blot. Uncropped blots are shown in Extended Data Figure 4-1.

### RNAscope and immunofluorescence

For RNAscope experiments, mice were deeply anesthetized with a mixture of ketamine/xylazine (87.5 and 12.5 mg/kg, respectively) and immediately perfused intracardially with PBS followed by 4% paraformaldehyde (Panreac, #252931.1211). After perfusion, brains were removed and postfixed overnight in the same solution, cryoprotected by immersion in 10, 20, 30% gradient sucrose (24 h for each sucrose gradient) at 4°C, and then embedded in OCT. Serial coronal cryostat sections (15 μm thick) through the whole brain were collected in microscope glass slides (Thermo Fisher Scientific, #J1800AMNZ) and stored at −80°C. RNAscope assay (Advanced Cell Diagnostics) was performed using RNAscope Intro Pack for Multiplex Fluorescent Reagent Kit v2 (#323136) with the mouse *Gap43* probe (#318621) following the manufacturer's instructions.

For immunofluorescence experiments, cryoprotected brains were mounted on standard cryomold with OCT, and serial coronal cryostat sections (30 µm thick) through the whole brain were collected in homemade antifreezing solution as free-floating sections and stored at −20°C. Slices were permeabilized and blocked in PBS containing 0.25% Triton X-100 and 10% goat serum (Pierce Biotechnology) for 1 h at room temperature. For the analysis of c-Fos-positive cells, an antigen retrieval step was conducted prior to the blocking step, incubating the slices in 10 mM sodium citrate, 0.05% Tween 20, pH 6.0, at 65°C for 30 min. Primary antibodies were diluted directly into the blocking buffer and incubated overnight at 4°C with the following dilutions: mouse anti-*pan*-GAP43 (1:1,000, SCBT #33705), rabbit anti-vGluT1 (1:500, Synaptic Systems, #135303), rabbit anti-c-Fos (1:750, SCBT #52). After three washes with PBS for 10 min, samples were subsequently incubated for 2 h at room temperature with the appropriate highly cross-adsorbed Alexa Fluor secondary antibody [Alexa Fluor 647 goat anti-rabbit IgG (1:1,000, Invitrogen #A-21244) or Alexa Fluor 647 goat anti-mouse IgG (1:1,000, Invitrogen, #A-21235)], together with DAPI (Roche) to visualize nuclei. After washing three times in PBS, sections were mounted onto microscope slides using Mowiol mounting media.

Immunofluorescence staining and hybridization images were acquired on an SP8 confocal microscope (Leica Microsystems) using LAS-X software version 3.4.2.18368. All quantifications were obtained from a minimum of three fields per section and three sections per condition. Fluorescent area was measured using Fiji ImageJ open-source software, establishing a threshold to measure only specific signal that was kept constant along the different images. Regions of interest were defined for cortical areas, basal ganglia nuclei, amygdala, as well as *cornu ammonis* 1 (CA1) and 3 (CA3) pyramidal layers, hilus, and granule cell layer of the dentate gyrus within the hippocampal formation. c-Fos-positive neurons within each region of interest were identified by the software as particles between 10 and 50 μm^2^, and the area of the region was calculated. Values were then averaged, and c-Fos density was calculated as the number of c-Fos-positive cells/mm^2^. Controls were included to ensure that none of the secondary antibodies produced any significant signal in preparations incubated in the absence of the corresponding primary antibodies. Representative images for each condition were prepared for figure presentation by applying brightness, contrast, and other adjustments uniformly.

### Electrophysiology

Mice were deeply anaesthetized with isoflurane and decapitated. The brains were removed from the skull and placed in ice-cold modified artificial CSF (aCSF), saturated with 95% O_2_ and 5% CO_2_, and containing the following (in mM): 230 sucrose, 10 MgSO_4_·7H_2_O, 2.5 KCl, 1.25 NaH_2_PO_4_·H_2_O, 0.5 CaCl_2_·H_2_O, 26 NaHCO_3_, and 10 d-glucose. Brains were sectioned into 300-µm-thick coronal slices using a vibratome (VT-1200S; Leica Microsystems). Slices were then immediately transferred to aCSF containing the following (in mM): 126 NaCl, 2.5 KCl, 1.25 NaH_2_PO_4_·H_2_O, 2 CaCl_2_·H_2_O, 2 MgSO_4_·7H_2_O, 26 NaHCO_3_, 10 d-glucose, 5 ʟ-glutathione, and 1 sodium pyruvate (gassed with 95% O_2_ and 5% CO_2_). Slices were incubated for 1 h at 32°C in this solution and then maintained at room temperature in the same solution until recording. Striatal slices were transferred to a recording chamber, continuously superfused with oxygenated aCSF at a rate of 2 ml/min at 32°C. Striatal neurons were visualized using infrared gradient contrast video microscopy (E600FN, Eclipse workstation, Nikon) and a water-immersion objective (Nikon Fluor 60×/1.0 NA). Medium spiny neurons (MSNs) were distinguished from interneurons by their morphology. Recordings from individual MSNs of the dorsal striatum were made using patch pipettes (5–8 MΩ) pulled from thick-walled borosilicate glass capillaries (G150-4; Warner Instruments) on a micropipette puller (P-97, Sutter Instruments). The patch pipette internal solution contained the following (in mM): 135 K-gluconate, 5 KCl, 5 NaCl, 2 MgCl_2_·6H_2_O, 10 HEPES, 0.05 CaCl_2_, 0.1 Na_4_EGTA, 0.4 Na_2_GTP, 2 Mg-ATP, and 5.3 biocytin. The osmolarity and pH of the intrapipette solution were adjusted at 290 mOsm and 7.2, respectively. Data were acquired using a MultiClamp 700B amplifier (Molecular Devices) controlled by pClamp 10 software (Molecular Devices), filtered at 4 kHz, and digitized at 20 kHz (Digidata 1550B). Series resistance of the cells was measured by injection of successive voltage steps of −5 mV. Data were discarded if the value changed >20% during the recording. Activation of cortical afferents was achieved using a bipolar stimulation electrode (SNEX-200; Phymep) placed just above the corpus callosum (stimulation intensity between 100 and 800 µA). These experiments were conducted in the presence of GABAergic transmission blockers (5 µM GBZ and 1 µM CGP55845). Double stimulations of 0.1 ms duration at a frequency of 20 Hz were performed to measure the amplitude of excitatory postsynaptic currents (EPSCs). Paired-pulse ratios (PPRs) were measured by dividing the peak of the second EPSC by the first EPSC. For these experiments, neurons were recorded at a potential of −70 mV. For corticostriatal induction of endocannabinoid-mediated long-term depression (LTD), the group-I metabotropic glutamate receptor (mGluRI) agonist (*S*)-3,5-dihydroxy-phenylglycine (DHPG) was applied at 50 µM for 10 min, following 5 min of baseline recording.

For input–output curves, PPR, and estimation of readily releasable pool (RRP) size experiments, the patch pipette internal solution contained the following (in mM): 135 cesium methanesulfonate, 5 KCl, 0.1 EGTA, 10 HEPES, 2 NaCl, 5 Mg-ATP, 0.4 Na-GTP, 10 Na_2_-phosphocreatine (pH 7.2 adjusted with CsOH, osmolarity 290 mOsm). To record glutamatergic (EPSCs) and GABAergic inputs (IPSCs) onto dorsal-striatum MSNs, the potential was clamped, respectively, at −70 and 0 mV (close to the reversal potential of glutamatergic receptors to minimize the influence of AMPA and NMDA receptor currents and allowing Cl^−^ flow through GABA_A_ receptors). To obtain input–output curves, an average of 10 consecutive EPSC/IPSCs were delivered at 0.2 Hz in response to increasing stimulation and normalized to responses at 400 mA. For PPR experiments, an average of 10 consecutive paired stimuli delivered at 0.2 Hz at different interstimulus interval were analyzed (dividing the peak of the second EPSC by the first EPSC). The size of the RRP was estimated as described previously (Martín et al., 2018, 2020). A tetanic train was applied (100 stimuli at 40 Hz), the cumulative EPSC amplitudes during this train were plotted, and the *y*-intercept was extended from the linear part of the curve (over times >1.5 s, when the cumulative amplitude curve reached steady state) and used to estimate the RRP size. The slope of this fitted plot was used to estimate the RRP replenishment rate, and the mean probability of vesicle release (Pves) was obtained by dividing the average of 10 consecutive EPSCs before the 40 Hz train by the estimated RRP size in each cell.

### DREADD-mediated neuronal manipulation in vivo

Eight-week-old Glu-GAP43^−/−^ mice and control GAP43^fl/fl^ littermates were injected stereotaxically with AAV8-hSyn-DIO-hM4Di-mCherry (Oliveira da Cruz et al., 2020) in 1.5 μl of PBS solution, aimed to target the motor cortex projecting onto the dorsal striatum. Each animal received two bilateral injections at the following coordinates (in mm to bregma): anteroposterior +1.5, lateral ±1.2, dorsoventral −1.7; and anteroposterior −0.5, lateral ±1.2, dorsoventral −1.2. Three weeks after surgery, mice were assigned to different experimental groups and injected intraperitoneally acutely with saline vehicle or clozapine-*N*-oxide (CNO; 2 mg/kg body weight). Open-field and RotaRod performance was analyzed 45 min postinjection as described above. In the case of the RotaRod test, data from trials 7–9 (Day 3; viz., the day of the injections) were pooled and averaged for each animal. Mice were subsequently sacrificed by intracardial perfusion, and their brains were excised for immunofluorescence analyses.

### Experimental design and statistical analyses

Data are presented as mean ± SEM. All datasets were tested for normality (Kolmogorov–Smirnov's test) and homoscedasticity (Fisher's *F* test and Levene's test) prior to analysis. The statistical test applied to each dataset is indicated in the corresponding figure legend. The precise *p* values are given in the figures. For clarity, only *p* values lower than 0.05 were considered statistically significant. All datasets are presented as dot plots. In all experiments, mice were allocated randomly into the different groups. Animal experiments were routinely performed and analyzed in a blinded manner for mouse genotype, viral injection, and pharmacological treatment (typically, an experimenter prepared the animals and their derived samples, and another experimenter conducted the assays blinded to group allocation). The sample size for each experiment was estimated based on previous studies conducted by our laboratory using similar brain-sample and animal-behavior approaches. The number of biological replicates (e.g., number of mice) is provided in the corresponding figure legends. The number of technical replicates (e.g., number of behavioral trials per mouse) is provided in the corresponding Materials and Methods. No data were excluded for the statistical analyses except when, very rarely, it was obvious that a technical problem had occurred in the measure. Source data from animal experiments were collected and analyzed as disaggregated for sex. No statistically significant differences were found between male and female mice in the numerous parameters measured in the study. Nonetheless, we note that, in some instances, sample size was not sufficiently high to enable solid post hoc statistical conclusions on potential sex differences. Graphs and statistics were generated by GraphPad Prism v8.0.1 (GraphPad Software).

## Results

### Selective deletion of GAP43 from glutamatergic neurons causes novelty-induced hyperactivity

**Figure 1.**
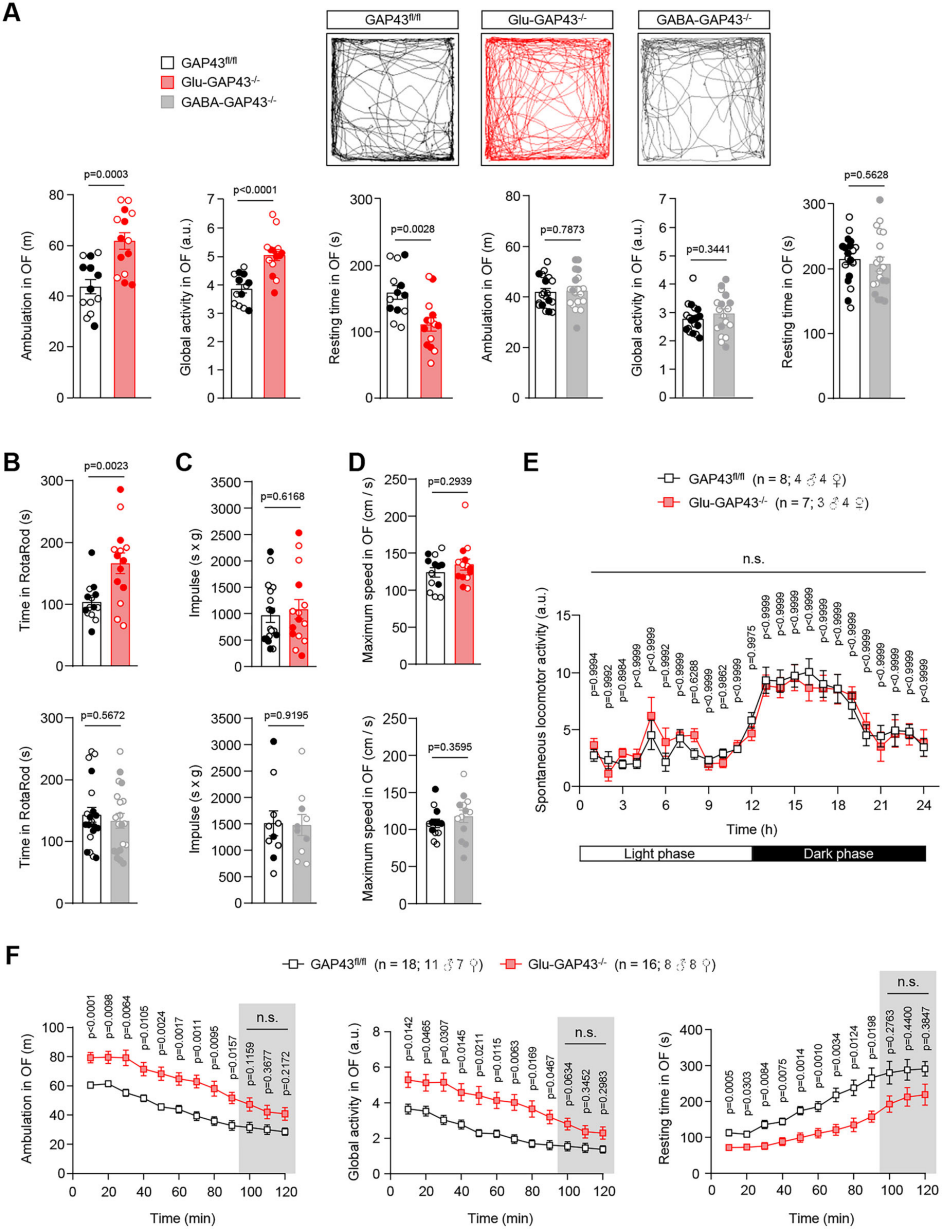
Glu-GAP43^−/−^ mice show novelty-induced hyperactivity. ***A***, Ambulation (total distance traveled, m), global activity (a.u.), and resting time (s) in a 10 min open-field (OF) test in Glu-GAP43^−/−^ mice and their GAP43^fl/fl^ littermates (left) and in GABA-GAP43^−/−^ mice and their GAP43^fl/fl^ littermates (right). Representative trajectory maps for each genotype are shown (means ± SEM; *n* = 13–19 mice per group; males, empty circles; females, filled circles; *p* values obtained by unpaired Student's *t* test). ***B***, RotaRod performance (time to fall, s) in the different genotypes (means ± SEM, *n* = 14–20 mice per group; males, empty circles; females, filled circles; *p* values obtained by unpaired Student's *t* test). ***C***, Holding impulse assessing muscle function and coordination (hanging time in s × body weight in g) in the different genotypes (means ± SEM, *n* = 10–18 mice per group; males, empty circles; females, filled circles; *p* values obtained by unpaired Student's *t* test). ***D***, Maximum speed (cm/s) in the OF test in the different genotypes (means ± SEM, *n* = 13–14 mice per group; males, empty circles; females, filled circles; *p* values obtained by unpaired Student's *t* test). ***E***, In-home cage spontaneous locomotor activity in the different genotypes over a 24 h light/dark period (means ± SEM; *n* = 7–8 mice per group; number of males and females indicated in the figure; *p* values obtained by two-way ANOVA with Sidak's multiple-comparisons test). ***F***, Ambulation (total distance traveled, m), global activity (a.u.), and resting time (s) in Glu-GAP43^−/−^ mice and their GAP43^fl/fl^ littermates over a 2 h period in an OF. The differences in the three parameters disappeared in the last 30 min, as highlighted by the shaded areas (means ± SEM; *n* = 16–18 mice per group; *n* of males and females is indicated; *p* values obtained by two-way ANOVA with Sidak's multiple-comparisons test).

We have previously characterized hippocampal excitability-related phenotypic alterations in Glu-GAP43^−/−^ and GABA-GAP43^−/−^ mice (Maroto et al., 2023). Here, we performed an additional set of behavioral tests and found that Glu-GAP43^−/−^ mice show an overt hyperlocomotor phenotype compared with their control GAP43^fl/fl^ littermates in a 10 min open-field test (Fig. 1*A*, left; GAP43^fl/fl^, *n* = 13 mice; Glu-GAP43^−/−^, *n* = 14 mice), which was reflected in an increased ambulation (*t*_(25) _= 4.150; *p* = 0.0003) and global activity (*t*_(25) _= 4.803; *p* < 0.0001), as well as a decreased resting time (*t*_(25) _= 3.319; *p* = 0.0028). In line with this hyperactive phenotype, Glu-GAP43^−/−^ mice displayed a better performance than their control littermates in the RotaRod test (Fig. 1*B*, top; *n* = 14 mice per group; *t*_(26) _= 3.3770; *p* = 0.0023), which may also point to the occurrence of repetitive and stereotyped routines related to autistic-like behaviors (Rothwell et al., 2014). In contrast, GABA-GAP43^−/−^ mice did not show any difference from their control littermates in these behavioral tests (Fig. 1*A*, right; ambulation: *n* = 19 mice per group; *t*_(36) _= 0.2718, *p* = 0.7873; global activity: *n* = 19 mice per group; *t*_(36) _= 0.9588, *p* = 0.3441; resting time: *n* = 19 mice per group; *t*_(36) _= 0.5840, *p* = 0.5628; Fig. 1*B*, bottom; *n* = 20 mice per group; *t*_(38) _= 0.5772, *p* = 0.5672). Of note, none of the two knock-out mouse lines displayed alterations in muscle strength (Fig. 1*C*, top; GAP43^fl/fl^, *n* = 18 mice, Glu-GAP43^−/−^, *n* = 15 mice; *t*_(31) _= 0.5054, *p* = 0.6168; Fig. 1*C*, bottom; GAP43^fl/fl^ and GABA-GAP43^−/−^, *n* = 10 mice per group; *t*_(18) _= 0.1025, *p* = 0.9195) and in the maximum speed reached in the open-field test (Fig. 1*D*, top; GAP43^fl/fl^, *n* = 13 mice, Glu-GAP43^−/−^, *n* = 14 mice; *t*_(25) _= 0.1072, *p* = 0.2939; Fig. 1*D*, bottom; GAP43^fl/fl^ and GABA-GAP43^−/−^, *n* = 13 mice per group; *t*_(24) _= 0.9342, *p* = 0.3595).

To analyze the hyperlocomotor phenotype of Glu-GAP43^−/−^ mice in further detail, we measured the spontaneous locomotor activity of the animals in their home cages during the complete circadian cycle (12 h of darkness and 12 h of light) for 3 consecutive days and averaged the triplicate values of each time point for each animal (Fig. 1*E*). Glu-GAP43^−/−^ and GAP43^fl/fl^ mice showed a similar diurnal/nocturnal activity profile, with no significant change at any time point measured (GAP43^fl/fl^, *n* = 8 mice; Glu-GAP43^−/−^, *n* = 7 mice; *F*_(1,13) _= 0.0329, individual *p* values shown in Fig. 1*E*), thus strongly suggesting that the hyperactivity shown by Glu-GAP43^−/−^ mice in the open-field test is induced by the exposure to a novel environment. Hence, the ambulation of Glu-GAP43^−/−^ and GAP43^fl/fl^ mice in the open-field test was registered over a longer period (2 h) and analyzed in 10 min bins (Fig. 1*F*). Glu-GAP43^−/−^ mice were significantly more active than GAP43^fl/fl^ controls for the first 90 min, but their activity returned to control levels thereafter (GAP43^fl/fl^, *n* = 18 mice; Glu-GAP43^−/−^, *n* = 16 mice; ambulation: *F*_(1,33) _= 22.53; global activity: *F*_(1,33) _= 16.24; resting time: *F*_(1,33) _= 16.13; individual *p* values shown in Fig. 1*F*). Taken together, these data indicate that the hyperactive phenotype observed in Glu-GAP43^−/−^ mice is triggered by their exposure to a novel environment and, once the animals are familiarized with their surroundings, their activity declines to baseline, control levels.

### Selective deletion of GAP43 from glutamatergic neurons induces hyperactivity-associated behavioral deficits

**Figure 2.**
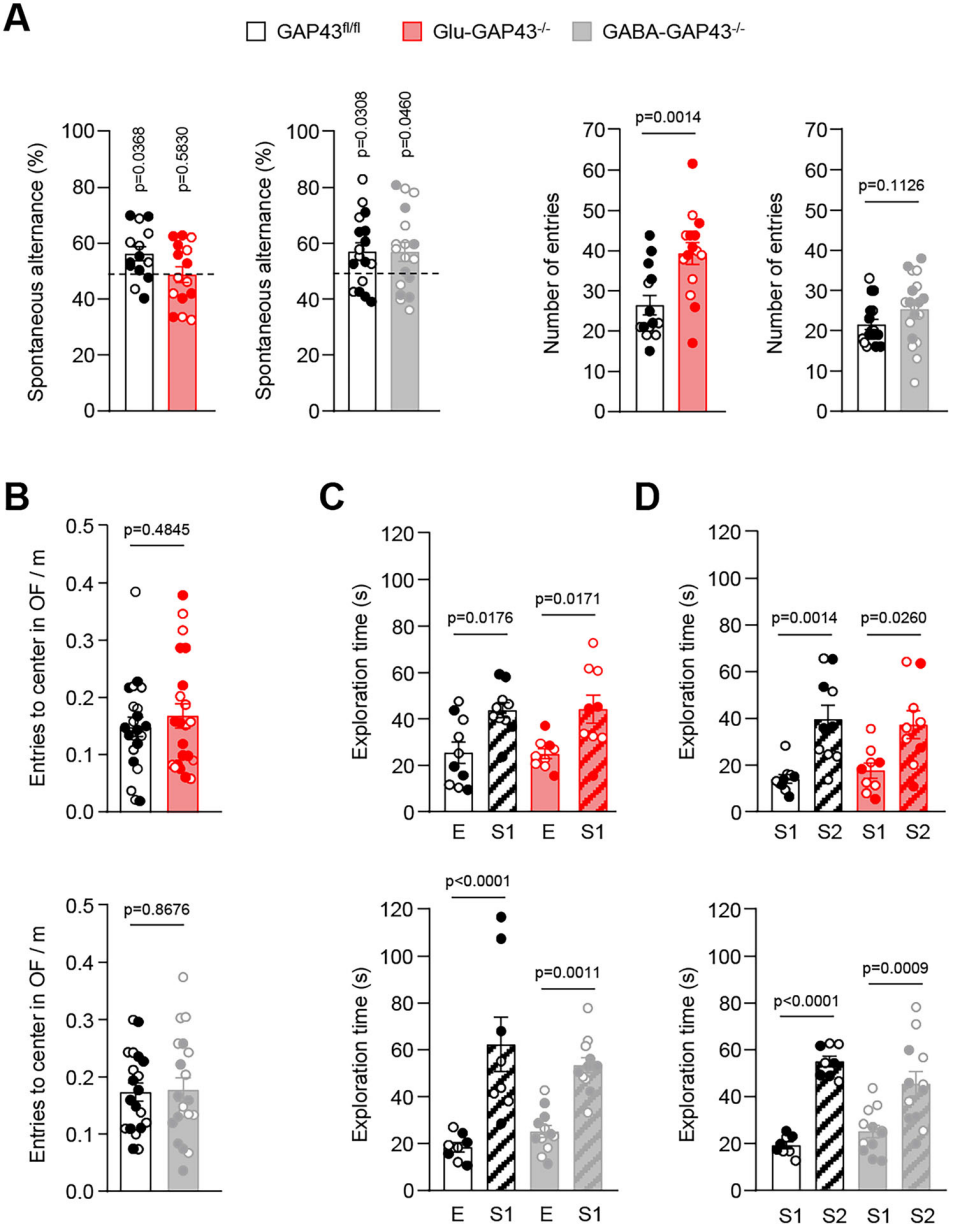
Glu-GAP43^−/−^ mice show alterations in other hyperactivity-associated behavioral parameters. ***A***, Percentage of spontaneous alternation (left) and total number of arm entries (right) in Glu-GAP43^−/−^ mice and their GAP43^fl/fl^ littermates and in GABA-GAP43^−/−^ mice and their GAP43^fl/fl^ littermates (means ± SEM, *n* = 14–18 mice per group; males, empty circles; females, filled circles; *p* values obtained by one-sample *t* test or unpaired Student's *t* test). ***B***, Anxiety-like behavior expressed as normalized entries to the center of the arena in the open-field (OF) test in Glu-GAP43^−/−^ mice and their GAP43^fl/fl^ littermates (top) and GABA-GAP43^−/−^ mice and their GAP43^fl/fl^ littermates (bottom; means ± SEM, *n* = 19–22 mice per group; males, empty circles; females, filled circles; *p* values obtained by unpaired Student's *t* test). ***C***, ***D***, Sociability (***C***), expressed as the preference for Subject 1 (S1) over an empty cage (E), and social novelty preference (***D***), expressed as the preference for a new Subject 2 (S2) over a familiar S1, in Glu-GAP43^−/−^ mice and their GAP43^fl/fl^ littermates (top), and GABA-GAP43^−/−^ mice and their GAP43^fl/fl^ littermates (bottom, means ± SEM, *n* = 8–12 mice per group; males, empty circles; females, filled circles; *p* values obtained by one-way ANOVA with Tukey's multiple-comparisons test).

Next, we conducted a Y-maze test, which relies on the innate curiosity of the animal to explore arms that they have not been previously visited, thus assessing short-term spatial memory, habituation to recently experienced stimuli and responsiveness to novelty (Sanderson and Bannerman, 2012). The percentage of triads of arm entries in which the mouse sequentially visited each possible arm without repeating, expressed as spontaneous alternance (Fig. 2*A*, left), as well as the total number of arm entries (Fig. 2*A*, right), were analyzed. Glu-GAP43^−/−^ mice had an impaired performance in this task, as they showed a spontaneous alternance of ∼50%, i.e., a “chance” level (*n* = 15 mice; *t*_(14) _= 0.5621; *p* = 0.5830). In contrast, both their GAP43^fl/fl^ littermates (*n* = 14 mice; *t*_(13) _= 2.326; *p* = 0.0368) and the GABA-GAP43^−/−^ animals (GAP43^fl/fl^: *n* = 17 mice; *t*_(16) _= 2.368; *p* = 0.0308; GABA-GAP43^−/−^: *n* = 18 mice; *t*_(17) _= 2.153, *p* = 0.0460) showed a normal alternance, with values significantly above 50%. Likewise, only Glu-GAP43^−/−^ mice showed an increased number of total arm entries compared with their GAP43^fl/fl^ littermates, in agreement with the aforementioned hyperlocomotor phenotype (Glu-GAP43^−/−^ vs GAP43^fl/fl^, *t*_(27) _= 3.549, *p* = 0.0014; GABA-GAP43^−/−^ vs GAP43^fl/fl^, *t*_(34) _= 1.629, *p* = 0.1126).

Regarding anxiety-like behaviors, we found no differences in the number of entries to the central part of the open-field arena as normalized to the total distance traveled for either Glu-GAP43^−/−^ mice (Fig. 2*B*, top; *n* = 22 mice per group; *t*_(42) _= 0.7054, *p* = 0.4845) or GABA-GAP43^−/−^ mice (Fig. 2*B*, bottom; *n* = 19 mice per group; *t*_(36) _= 0.2805, *p* = 0.7807) compared with their GAP43^fl/fl^ littermates, in line with previous data obtained in the elevated plus maze test (Maroto et al., 2023). Additionally, we did not detect alterations of sociability in the mouse lines, as both Glu-GAP43^−/−^, GABA-GAP43^−/−^, and GAP43^fl/fl^ mice displayed a significant preference for exploring a compartment with an unfamiliar juvenile mouse (stranger 1, S1) rather than an empty compartment (empty, E; Fig. 2*C*, top; Glu-GAP43^−/−^ *n* = 9 mice, GAP43^fl/fl^ *n* = 10 mice; *F*_(3,34) _= 6.582; Glu-GAP43^−/−^ S1 vs Glu-GAP43^−/−^ E, *p* = 0.0171; GAP43^fl/fl^ S1 vs GAP43^fl/fl^ E, *p* = 0.0176; Fig. 2*C*, bottom; GABA-GAP43^−/−^, *n* = 12 mice; GAP43^fl/fl^, *n* = 8 mice; *F*_(3,36) _= 14.87; GABA-GAP43^−/−^ S1 vs GABA-GAP43^−/−^ E, *p* = 0.0011; GAP43^fl/fl^ S1 vs GAP43^fl/fl^ E, *p* < 0.0001). Preference for social novelty was neither altered, as each mouse line showed a significant preference for a novel stranger mouse (stranger 2, S2) than for S1 (Fig. 2*D*, top; Glu-GAP43^−/−^, *n* = 9 mice; GAP43^fl/fl^, *n* = 10 mice; *F*_(3,34) _= 8.538; Glu-GAP43^−/−^ S1 vs Glu-GAP43^−/−^ S2, *p* = 0.0260; GAP43^fl/fl^ S1 vs GAP43^fl/fl^ S2, *p* = 0.0014; Fig. 2*D*, bottom; GABA-GAP43^−/−^, *n* = 12 mice; GAP43^fl/fl^, *n* = 8 mice; *F*_(3,36) _= 18.36; GABA-GAP43^−/−^ S1 vs GABA-GAP43^−/−^ S2, *p* = 0.0009; GAP43^fl/fl^ S1 vs GAP43^fl/fl^ S2, *p* < 0.0001). Taken together, these data indicate that Glu-GAP43^−/−^ mice display hyperactivity-associated deficits in short-term memory and habituation to newly perceived stimuli, which might involve a temporary reduction in attention paid to those stimuli, with no changes in traits related to anxious or social behaviors.

### Selective deletion of GAP43 from glutamatergic neurons overactivates striatal neurons upon exposure to a novel environment

**Figure 3.**
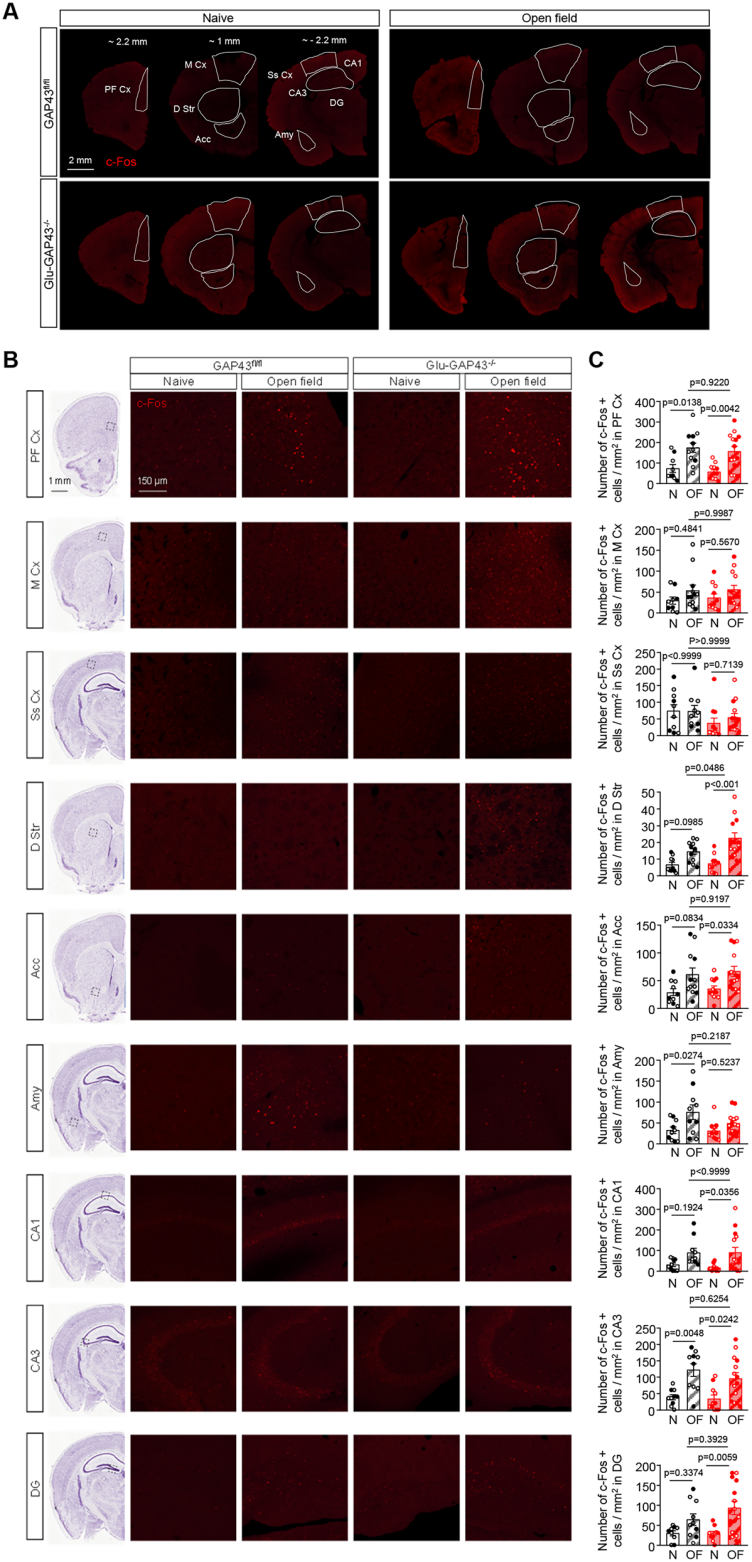
Glu-GAP43^−/−^ mice show a striatal-neuron overactivation upon exposure to a novel environment. ***A***, c-Fos immunostaining in the brain is shown in representative brain slices from GAP43^fl/fl^ and Glu-GAP43^−/−^ mice, either naive or after a novel open-field (OF) exposure. The brain regions in which c-Fos-immunopositive cells were analyzed are delimited with a solid white line. ***B***, Left, Black dashed-line boxes on representative brain coronal hemi-sections delimiting the regional location of the high-magnification insets shown. Image credit: Allen Institute. Right, Representative c-Fos high-magnification immunofluorescence images of each analyzed region. ***C***, Quantification of the number of c-Fos-positive cells/mm^2^ in each of the analyzed areas (means ± SEM, *n* = 9–14 mice per group; males, empty circles; females, filled circles; *p* values obtained by two-way ANOVA with Tukey's multiple-comparisons test). PF Cx, prefrontal cortex; M Cx, motor cortex; Ss Cx, somatosensory cortex; D Str, dorsal striatum; Acc, nucleus accumbens; Amy, amygdala; CA1, c*ornu ammonis* 1; CA3, c*ornu ammonis* 3; DG, dentate gyrus.

To elucidate the anatomical pattern of neuronal activation in Glu-GAP43^−/−^ mice upon exposure to a novel environment, we immunolabeled c-Fos protein expression (Hunt et al., 1988; Ceccatelli et al., 1989; Sheng and Greenberg, 1990) in Glu-GAP43^−/−^ and GAP43^fl/fl^ mice after a novel 30 min open-field test, together with the respective control group of “naive” mice of each genotype, which did not perform the test. We mapped the expression of c-Fos in different brain areas (Fig. 3*A*; Fig. 3*B*, insets) and observed an induction after the open-field test in most areas analyzed [Fig. 3*C*; prefrontal cortex: GAP43^fl/fl^ naive, *n* = 9 mice, GAP43^fl/fl^ open field (OF), *n* = 12 mice, Glu-GAP43^−/−^ naive, *n* = 12 mice, Glu-GAP43^−/−^ OF, *n* = 14 mice, *F*_(1,43) _= 22.89; motor cortex: GAP43^fl/fl^ naive, *n* = 9 mice, GAP43^fl/fl^ OF, *n* = 12 mice, Glu-GAP43^−/−^ naive, *n* = 11 mice, Glu-GAP43^−/−^ OF, *n* = 14 mice, *F*_(1,42) _= 3.759; somatosensory cortex: GAP43^fl/fl^ naive, *n* = 10 mice, GAP43^fl/fl^ OF, *n* = 10 mice, Glu-GAP43^−/−^, naive *n* = 11 mice, Glu-GAP43^−/−^ OF, *n* = 14 mice, *F*_(1,41) _= 0.2186; dorsal striatum: GAP43^fl/fl^ naive, *n* = 10 mice, GAP43^fl/fl^ OF, *n* = 12 mice, Glu-GAP43^−/−^ naive, *n* = 12 mice, Glu-GAP43^−/−^ OF, *n* = 13 mice, *F*_(1,43) _= 27.09; nucleus accumbens: GAP43^fl/fl^ naive, *n* = 10 mice, GAP43^fl/fl^ OF, *n* = 12 mice, Glu-GAP43^−/−^ naive, *n* = 12 mice, Glu-GAP43^−/−^ OF, *n* = 14 mice, *F*_(1,44) _= 13.84; amygdala: GAP43^fl/fl^ naive, *n* = 10 mice, GAP43^fl/fl^ OF, *n* = 10 mice, Glu-GAP43^−/−^ naive, *n* = 12 mice, Glu-GAP43^−/−^ OF, *n* = 14 mice, *F*_(1,42) _= 9.617; hippocampal dentate gyrus: GAP43^fl/fl^ naive, *n* = 9 mice, GAP43^fl/fl^ OF, *n* = 10 mice, Glu-GAP43^−/−^ naive, *n* = 10 mice, Glu-GAP43^−/−^ OF, *n* = 14 mice, *F*_(1,39) _= 13.09; hippocampal CA1: GAP43^fl/fl^ naive, *n* = 10 mice, GAP43^fl/fl^ OF, *n* = 10 mice, Glu-GAP43^−/−^ naive, *n* = 11 mice, Glu-GAP43^−/−^ OF, *n* = 14 mice, *F*_(1,41) _= 11.55; hippocampal CA3: GAP43^fl/fl^ naive, *n* = 10 mice, GAP43^fl/fl^ OF, *n* = 10 mice, Glu-GAP43^−/−^ naive, *n* = 11 mice, Glu-GAP43^−/−^ OF, *n* = 14 mice, *F*_(1,43) _= 21.86; individual *p* values shown in Fig. 3*C*]. Remarkably, the novel open-field test-mediated increase in the number of c-Fos-positive neurons was only significantly higher in the dorsal striatum of Glu-GAP43^−/−^ mice compared with their GAP43^fl/fl^ littermates (Fig. 3*C*; *F*_(1,43) _= 27.09, GAP43^fl/fl^ OF vs GAP43^fl/fl^ naive, *p* = 0.0985; Glu-GAP43^−/−^ OF vs Glu-GAP43^−/−^ naive, *p* < 0.0001; *F*_(1,43) _= 3.702; Glu-GAP43^−/−^ OF vs GAP43^fl/fl^ OF, *p* = 0.0486; Glu-GAP43^−/−^ naive vs GAP43^fl/fl^ naive, *p* = 0.9993). Taken together, these data show that dorsal-striatum neurons are selectively overactivated in Glu-GAP43^−/−^ mice compared with their GAP43^fl/fl^ littermates upon the exposure to a novel environment, thus suggesting that an abnormal glutamatergic transmission in that brain region underlies the novelty-induced hyperactive phenotype observed in Glu-GAP43^−/−^ mice.

### GAP43 is highly expressed in corticostriatal terminals of the mouse brain

**Figure 4.**
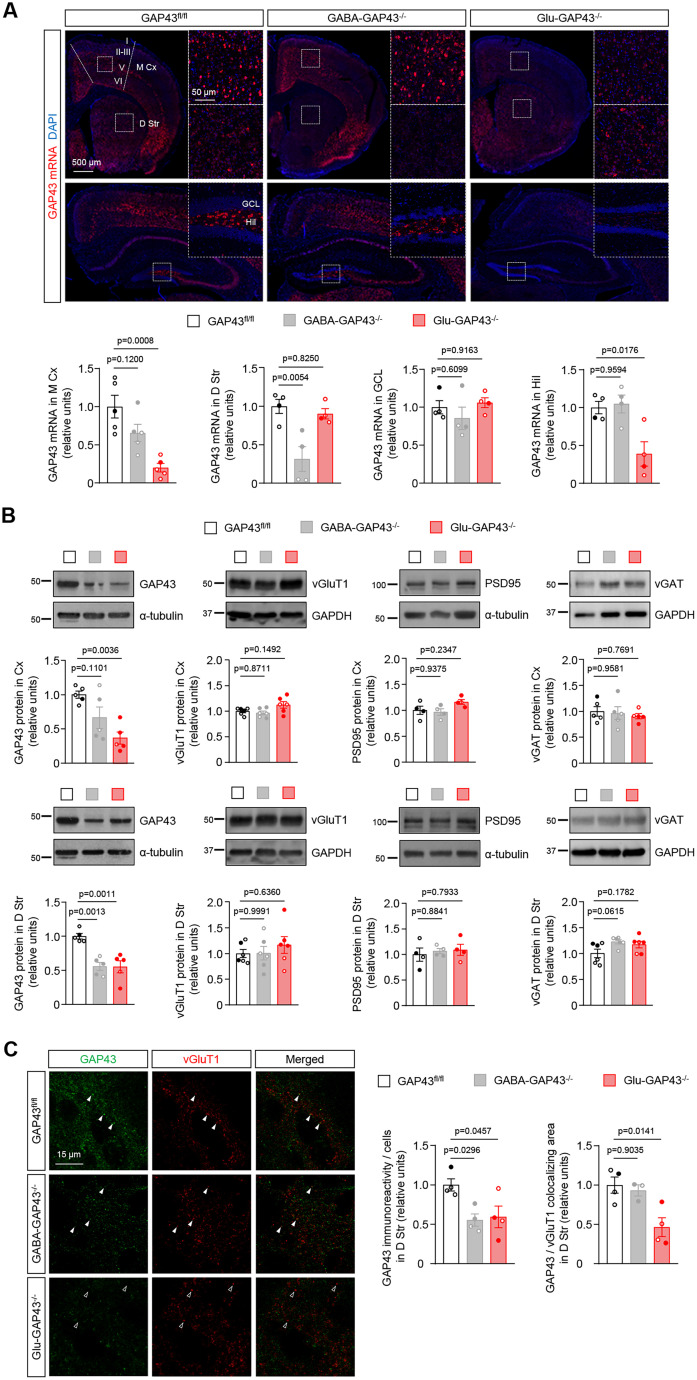
GAP43 is highly expressed in corticostriatal terminals of the mouse brain. ***A***, Top, Expression of GAP43 mRNA (in red) in the brain of GAP43^fl/fl^, GABA-GAP43^−/−^, and Glu-GAP43^−/−^ mice. Nuclei were stained with DAPI (blue). The dotted line depicts the high-magnification inset shown aside. Representative images are shown. Bottom, Quantification of GAP43 mRNA levels in the cortex, dorsal striatum, hippocampal granular cell layer, and hilus of the hippocampal dentate gyrus (means ± SEM; *n* = 4–5 mice per group; males, empty circles; females, filled circles; *p* values obtained by one-way ANOVA with Tukey's multiple-comparisons test). ***B***, Representative Western blots of GAP43, vGluT1, PSD95 and vGAT proteins in the cortex and dorsal striatum of GAP43^fl/fl^, GABA-GAP43^−/−^, and Glu-GAP43^−/−^ mice. Quantification of normalized optical density values of each protein relative to those of the loading control are shown below each representative blot (means ± SEM; *n* = 4–6 mice per group; males, empty circles; females, filled circles; *p* values obtained by one-way ANOVA with Tukey's multiple-comparisons test). ***C***, Left, Representative images of GAP43 and vGluT1 immunolocalization in the dorsal striatum of GAP43^fl/fl^, GABA-GAP43^−/−^, and Glu-GAP43^−/−^ mice. Filled arrowheads point to representative colocalizing boutons, and empty arrowheads point to representative noncolocalizing boutons. Right, Quantification of GAP43 immunoreactivity and GAP43/vGluT1-colocalizing area in the dorsal striatum (means ± SEM; *n* = 3–4 mice per group; males, empty circles; females, filled circles; *p* values obtained by one-way ANOVA with Tukey's multiple-comparisons test). M Cx, motor cortex; D Str, dorsal striatum; GCL, granule cell layer; Hil, hilus; I-VI, cortical layers I to VI. See Extended Data Figure 4-1 for more details.

The striatum is composed almost exclusively of GABAergic MSNs, which receive glutamatergic projections mainly from the cortex (Kreitzer, 2009). To determine the expression of GAP43 in the mouse dorsal striatum, we first performed in situ hybridization experiments in brain sections of our mouse lines by RNAscope technology. GAP43 mRNA was found widely expressed in the brain of control GAP43^fl/fl^ mice. Specifically, high GAP43 mRNA levels were detected in the cortex, particularly in the projection neuron-enriched layer V, as well as in the striatum (Fig. 4*A*, left). In GABA-GAP43^−/−^ mice, GAP43 mRNA levels were strongly decreased in the dorsal striatum compared with GAP43^fl/fl^ littermates, while a slight, nonsignificant reduction was found in the cortex (Fig. 4*A*, middle). In Glu-GAP43^−/−^ mice, GAP43 mRNA levels were notably reduced in the cortex and the hippocampal formation, two regions enriched in glutamatergic neurons expressing the *Nex1* promoter (Kleppisch et al., 2003), but not in the dorsal striatum (Fig. 4*A*, right; Fig. 4*A*, bottom. Cortex: *n* = 5 mice per group; *F*_(2,12) _= 12.60; GABA-GAP43^−/−^ vs GAP43^fl/fl^, *p* = 0.1200, Glu-GAP43^−/−^ vs GAP43^fl/fl^, *p* = 0.0008. Dorsal striatum: *n* = 4 mice per group; *F*_(2,9) _= 10.60; GABA-Gap43^−/−^ vs GAP43^fl/fl^, *p* = 0.0054, Glu-GAP43^−/−^ vs GAP43^fl/fl^, *p* = 0.8250). We noted a high abundance of GAP43 mRNA in hilar glutamatergic (presumably mossy) cells and a virtual absence of GAP43 mRNA expression in the granule cell layer of the dentate gyrus, which were used as internal positive and negative control of GAP43 mRNA expression, respectively (Meberg and Routtenberg, 1991; Fig. 4*A*, bottom. Granule cell layer: *n* = 4 mice per group; *F*_(2,12) _= 1.000; GABA-GAP43^−/−^ vs GAP43^fl/fl^, *p* = 0.6099, Glu-Gap43^−/−^ vs GAP43^fl/fl^, *p* = 0.9163. Hilus: *n* = 4 mice per group; *F*_(2,9) _= 8.684; GABA-GAP43^−/−^ vs GAP43^fl/fl^, *p* = 0.9594, Glu-GAP43^−/−^ vs GAP43^fl/fl^, *p* = 0.0176).

10.1523/JNEUROSCI.0701-24.2024.f4-1 Fig 4-1 Uncropped version of the blots shown in Fig 4. Download Fig 4-1, TIF file.

Next, we measured GAP43 protein levels by Western blot in the cortex and dorsal striatum (Fig. 4*B*). Cortical GAP43 protein levels were notably reduced in Glu-GAP43^−/−^ mice and slightly, nonsignificantly reduced in GABA-GAP43^−/−^ mice, in line with the aforementioned changes in mRNA expression (*n* = 5 mice per group; *F*_(2,12) _= 8.590; GABA-GAP43^−/−^ vs GAP43^fl/fl^, *p* = 0.1101, Glu-GAP43^−/−^ vs GAP43^fl/fl^, *p* = 0.0036). In contrast, dorsal-striatum GAP43 protein levels were reduced not only in GABA-GAP43^−/−^ mice, as expected, but also in Glu-GAP43^−/−^ mice (*n* = 5 mice per group; *F*_(2,12) _= 15.19; GABA-GAP43^−/−^ vs GAP43^fl/fl^, *p* = 0.0013, Glu-GAP43^−/−^ vs GAP43^fl/fl^, *p* = 0.0011). The reduction of GAP43 protein levels in the dorsal striatum of Glu-GAP43^−/−^ and GABA-GAP43^−/−^ mice was also assessed by immunofluorescence (Fig. 4*C*; *n* = 4 mice per group; *F*_(2,9) _= 5.998; GABA-GAP43^−/−^ vs GAP43^fl/fl^, *p* = 0.0296, Glu-GAP43^−/−^ vs GAP43^fl/fl^, *p* = 0.0457). In the case of Glu-GAP43^−/−^ mice, but not of GABA-GAP43^−/−^ animals, this decrease was evident in glutamatergic terminals, as shown by colocalization with vGluT1 (Fig. 4*C*; GAP43^fl/fl^, *n* = 4 mice, GABA-GAP43^−/−^, *n* = 3 mice, Glu-GAP43^−/−^, *n* = 4 mice; *F*_(2,8) _= 8.041; GABA-GAP43^−/−^ vs GAP43^fl/fl^, *p* = 0.9035, Glu-GAP43^−/−^ vs GAP43^fl/fl^, *p* = 0.0141). Taken together, these data support a strong abundance of the GAP43 protein in the corticostriatal circuitry, particularly in glutamatergic terminals projecting from the cortex onto the dorsal striatum. Of note, protein levels of the synaptic glutamatergic markers vGluT1 and PSD95 and the synaptic GABAergic marker vGAT were not altered in either Glu-GAP43^−/−^ or GABA-GAP43^−/−^ mice compared with their GAP43^fl/fl^ littermates (Fig. 4*B*), thus suggesting that no gross structural synaptic alterations occur in the two knock-out lines (cortex; vGluT1: *n* = 6 mice per group; *F*_(2,15) _= 3.472; GABA-GAP43^−/−^ vs GAP43^fl/fl^, *p* = 0.8711, Glu-GAP43^−/−^ vs GAP43^fl/fl^, *p* = 0.1492; PSD95: *n* = 4 mice per group; *F*_(2,9) _= 2.562; GABA-GAP43^−/−^ vs GAP43^fl/fl^, *p* = 0.9375, Glu-GAP43^−/−^ vs GAP43^fl/fl^, *p* = 0.2347; vGAT: *n* = 5 mice per group; *F*_(2,12) _= 2.469; GABA-GAP43^−/−^ vs GAP43^fl/fl^, *p* = 0.9581, Glu-GAP43^−/−^ vs GAP43^fl/fl^, *p* = 0.7691; dorsal striatum; vGluT1: *n* = 6 mice per group; *F*_(2,15) _= 0.5417; GABA-GAP43^−/−^ vs GAP43^fl/fl^, *p* = 0.9991, Glu-GAP43^−/−^ vs GAP43^fl/fl^, *p* = 0.6360; PSD95: *n* = 4 mice per group; *F*_(2,9) _= 0.5792; GABA-GAP43^−/−^ vs GAP43^fl/fl^, *p* = 0.8841, Glu-GAP43^−/−^ vs GAP43^fl/fl^, *p* = 0.7933; vGAT: *n* = 5 mice per group; *F*_(2,15) _= 3.069; GABA-GAP43^−/−^ vs GAP43^fl/fl^, *p* = 0.0615, Glu-GAP43^−/−^ vs GAP43^fl/fl^, *p* = 0.1782).

### Selective deletion of GAP43 from glutamatergic neurons abrogates corticostriatal endocannabinoid-mediated long-term depression

**Figure 5.**
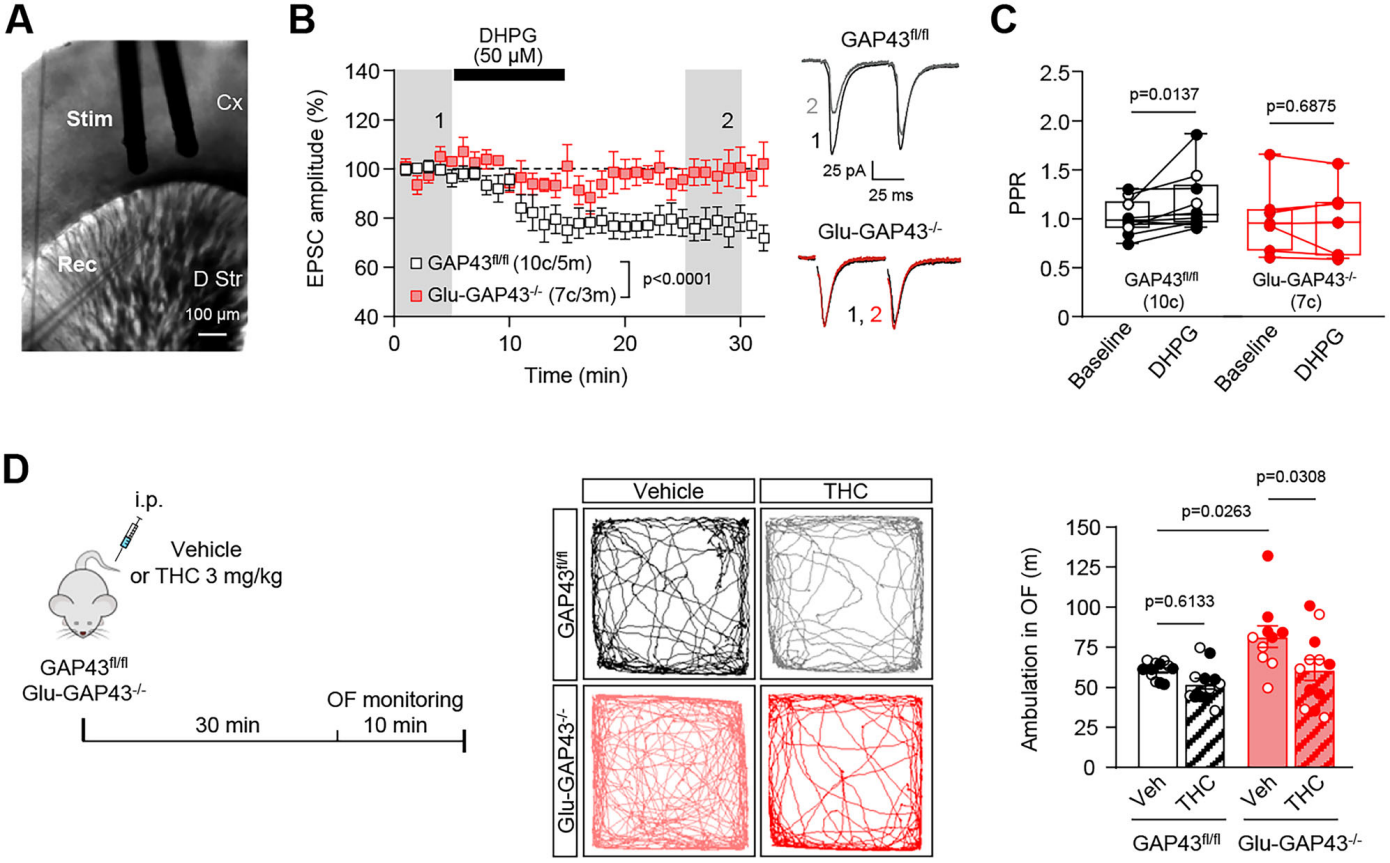
Glu-GAP43^−/−^ mice show an abrogation of corticostriatal endocannabinoid-mediated long-term depression. ***A***, Representative infrared differential interference contrast (DIC) image of a coronal corticostriatal slice showing the position of the stimulating electrode (Stim) and the recording electrode (Rec). Cx, cortex; D Str, dorsal striatum. ***B***, EPSCs recorded from MSNs of the dorsal striatum in whole-cell patch-clamp configuration upon DHPG bath application (50 μM, 10 min). Left, Time course summary plot of EPSC amplitudes, before and after DHPG bath application, in Glu-GAP43^−/−^ and GAP43^fl/fl^ mice (means ± SEM, c, cells; m, mice; shaded areas indicate the time intervals at which the statistical analysis was conducted; *p* values obtained by paired Student's *t* test for DHPG vs baseline or by unpaired Student's *t* test for Glu-GAP43^−/−^ vs GAP43^fl/fl^ mice). Right, Representative EPSC traces. ***C***, Quantification of PPR before (baseline) and after DHPG application in GAP43^fl/fl^ and Glu-GAP43^−/−^ mice (means ± SEM; c, cells; males, empty circles; females, filled circles; *p* values obtained by Wilcoxon's signed-rank test). ***D***, GAP43^fl/fl^ and Glu-GAP43^−/−^ mice were injected intraperitoneally with vehicle or THC (3 mg/kg), and 30 min later locomotor activity was analyzed (left). Representative trajectory maps of each condition (middle) together with ambulation (total distance traveled, m) in a 10 min open-field (OF) test (right; means ± SEM; *n* = 10–12 mice per group; males, empty circles; females, filled circles; *p* values obtained by two-way ANOVA with Tukey's multiple-comparisons test). See Extended Data Figure 5-1 for more details.

We subsequently examined whether corticostriatal synaptic plasticity was altered in Glu-GAP43^−/−^ mice. We stimulated layer V cortical neurons and performed whole-cell patch-clamp recordings in MSNs of the dorsal striatum (Fig. 5*A*). We first measured the basal amplitude of recorded EPSCs in the presence of GABAergic transmission blockers. As previously reported (Lovinger, 2010), endocannabinoid-mediated LTD of EPSCs was overtly induced by the mGluRI agonist DHPG in corticostriatal synapses of control GAP43^fl/fl^ mice (Fig. 5*B*, white symbols; 77.83 ± 2.35% of baseline, *n* = 10 cells; *t*_(4) _= 0.5583, *p* = 0.0002). This was associated with changes in the PPR (Fig. 5*C*; baseline: 1.02 ± 0.17, *n* = 10 cells; after DHPG: 1.17 ± 0.30, *n* = 10 cells; *W* = 47.00, *p* = 0.0137), thus supporting a mechanism mediated by presynaptic CB_1_Rs. In fact, we interrogated a mouse whole-brain scRNA-seq atlas (Yao et al., 2023) and found an overt coexpression of the mRNAs encoding GAP43 and CB_1_R in primary motor cortex projection neurons (Extended Data Fig. 5-1*A*). In contrast to control GAP43^fl/fl^ mice, bath-applied DHPG failed to trigger LTD in Glu-GAP43^−/−^ animals (Fig. 5*B*, red symbols; 99.08 ± 1.47% of baseline, *n* = 7 cells; *t*_(4) _= 13.92, *p* = 0.6064; *t*_(8) _= 17.57, Glu-GAP43^−/−^ vs GAP43^fl/fl^ after DHPG *p* < 0.0001) and did not change PPR in those animals (Fig. 5*C*; baseline: 0.99 ± 0.35, *n* = 7 cells; after DHPG: 0.95 ± 0.37, *n* = 7 cells; *W* = −6.00, *p* = 0.6875), which suggests that an overactive state of this synapse due to a hypofunction of CB_1_Rs occurs upon GAP43 deletion.

10.1523/JNEUROSCI.0701-24.2024.f5-1 Fig 5-1 **Single-cell transcriptomic analysis of *Gap43* and *Cnr1* mRNAs in mouse primary motor cortex and dorsal striatum.** The single-cell RNA sequencing data from the Whole Mouse Brain Transcriptomic Cell Type Atlas was accessed using the online tool provided by the Allen Brain Map initiative (https://knowledge.brain-map.org/abcatlas, accessed on 12/06/2024). The dataset was filtered by dissection region [either primary motor cortex (MOp) or dorsal striatum (STRd)] prior to plotting the corresponding Uniform Manifold Approximation and Projection (UMAP) maps. **A.** UMAP representation of MOp cells (∼248,000 individual cells) colored by (*i*) neurotransmitter type, (*ii*) cell class (doi: 10.1038/s41586-023-06812-z), (*iii*) *Gap43* mRNA expression (in counts per million bases, CPM), and (*iv*) *Cnr1* mRNA expression (in CPM). As inferred from the overlay of the UMAPs, coincidental neuronal classes co-expressing *Gap43* and *Cnr1* mRNAs include projection neurons (IT-ET Glu). **B.** UMAP representation of dorsal STRd cells (∼55,600 individual cells) colored by (*i*) neurotransmitter type, (*ii*) cell class (doi: 10.1038/s41586-023-06812-z), (*iii*) *Gap43* mRNA expression (in CPM), and (*iv*) *Cnr1* mRNA expression (in CPM). As inferred from the overlay of the UMAPs, coincidental neuronal classes co-expressing *Gap43* and *Cnr1* mRNAs include medium spiny neurons (CNU-LGE GABA). Neurotransmitter type: AcCh, cholinergic; DA, dopaminergic; GABA, GABAergic; Glu, glutamatergic; NA, noradrenergic. Cell class: Astro, astrocyte; CGE, caudal ganglionic eminence; CNU, cerebral nuclei; CR, Cajal–Retzius; CT, corticothalamic; CTX, cerebral cortex; Epen, ependymal; ET, extratelencephalic; HYa, anterior hypothalamic; IMN, immature neuron; IT, intratelencephalic; L6b, layer 6b; LGE, lateral ganglionic eminence; MGE, medial ganglionic eminence; NP, near-projecting; OB, olfactory bulb; Oligo, oligodendrocyte; OPC, oligodendrocyte precursor cell. Download Fig 5-1, TIF file.

To further assess the possible involvement of a CB_1_R hypofunction in the hyperactive phenotype of Glu-GAP43^−/−^ mice, we acutely injected GAP43^fl/fl^ and Glu-GAP43^−/−^ mice with either vehicle or the CB_1_R agonist THC at 3 mg/kg (i.p.), and locomotor activity was measured 30 min later (Fig. 5*D*, left). This dose of THC was subeffective in GAP43^fl/fl^ mice but fully rescued the hyperactive phenotype of Glu-GAP43^−/−^ animals (Fig. 5*D*, middle and right; GAP43^fl/fl^ vehicle, *n* = 12 mice, GAP43^fl/fl^ THC, *n* = 12 mice, Glu-GAP43^−/−^ vehicle, *n* = 10 mice, Glu-GAP43^−/−^ THC, *n* = 12 mice; *F*_(1,42) _= 8.580; Glu-GAP43^−/−^ vehicle vs GAP43^fl/fl^ vehicle, *p* = 0.0263, GAP43^fl/fl^ THC vs GAP43^fl/fl^ vehicle, *p* = 0.6133, Glu-GAP43^−/−^ THC vs Glu-GAP43^−/−^ vehicle, *p* = 0.0308). Taken together, all these data support that GAP43 plays a key role in safeguarding the endocannabinoid-mediated long-term plasticity of corticostriatal glutamatergic terminals.

### Selective deletion of GAP43 from glutamatergic neurons enhances glutamatergic transmission at corticostriatal terminals

**Figure 6.**
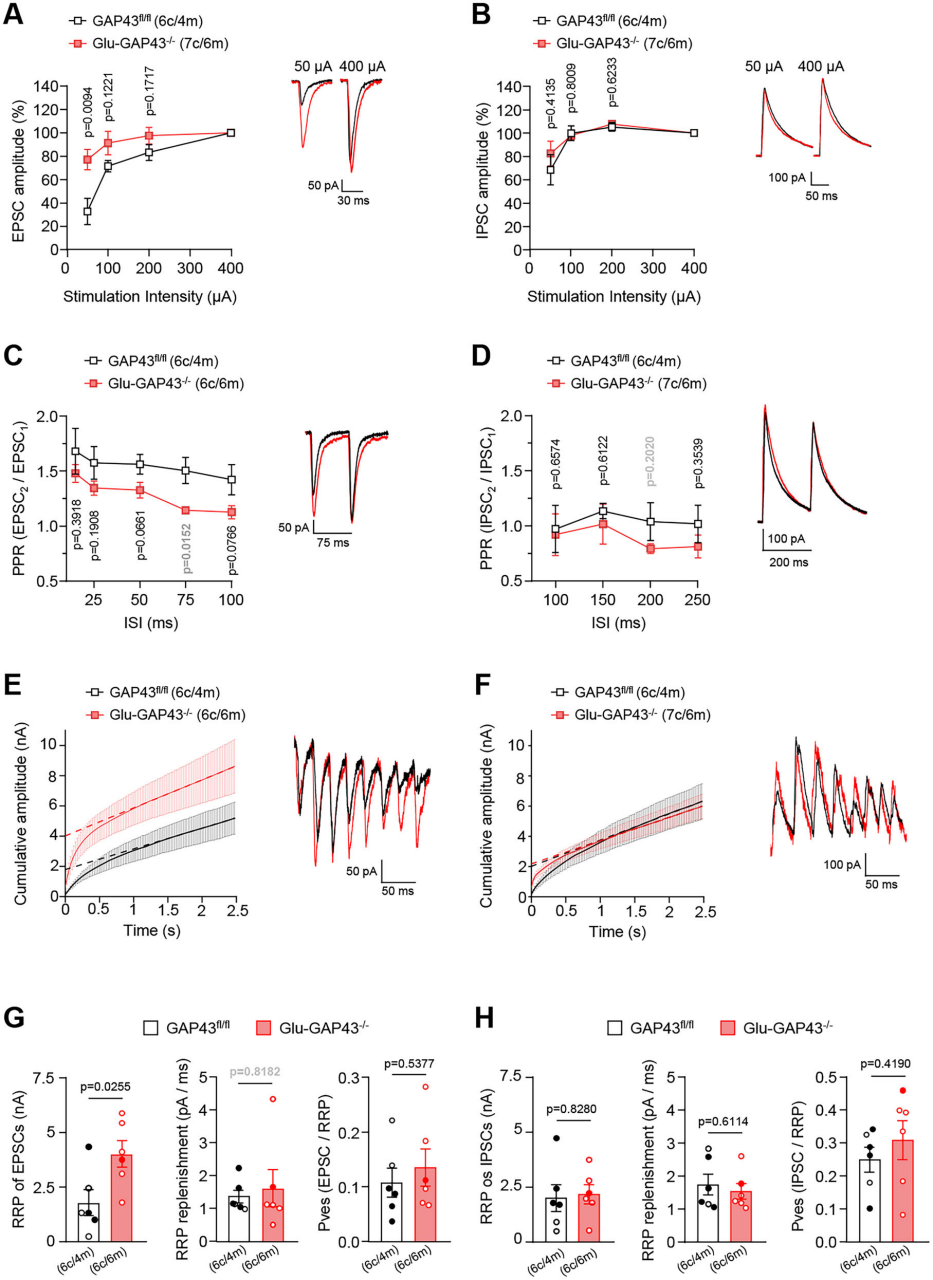
Glu-GAP43^−/−^ mice show an enhanced glutamatergic transmission at corticostriatal terminals. ***A***, ***B***, EPSC (***A***) and IPSC (***B***) amplitude recorded in the dorsal striatum upon increasing stimulation intensities. Left, Summary plot in Glu-GAP43^−/−^ and GAP43^fl/fl^ mice (means ± SEM, c, cells; m, mice; *p* values obtained by unpaired Student's *t* test). Right, Representative EPSC and IPSC traces obtained averaging 10 consecutive stimuli delivered at 0.2 Hz. ***C***, ***D***, PPR of the EPSCs (***C***) and IPSCs (***D***) in GAP43^fl/fl^ and Glu-GAP43^−/−^ mice at increasing interstimulus intervals (ISI). Left, Summary plot in Glu-GAP43^−/−^ and GAP43^fl/fl^ mice (means ± SEM; c, cells; *p* values obtained by unpaired Student's *t* test, shown in black; or Mann–Whitney's *U* test, shown in bold gray). Right, Representative EPSC and IPSC traces obtained averaging 10 consecutive paired stimuli delivered at 0.2 Hz. ***E***, ***F***, Left, Time course summary plot of EPSC (***E***) and IPSC (***F***) cumulative amplitudes in GAP43^fl/fl^ and Glu-GAP43^−/−^ mice during high-frequency trains (100 stimuli at 40 Hz). The *y*-intercept from a linear fit of the steady-state values is depicted. Right, Representative EPSC and IPSC traces of the first 8 stimuli of the train. ***G***, ***H***, Quantification of the RRP size, the replenishment rate (pA/ms), and the Pves (EPSC or IPSC amplitude value divided by the RRP size) of EPSCs (***G***) and IPSCs (***H***) in GAP43^fl/fl^ and Glu-GAP43^−/−^ mice (means ± SEM, c, cells; m, mice; males, empty circles; females, filled circles; *p* values obtained by unpaired Student's *t* test, shown in black; or Mann–Whitney's *U* test, shown in bold gray).

To further explore the possibility of a basal overactive state of corticostriatal presynaptic terminals in Glu-GAP43^−/−^ mice, we conducted analysis of input–output curves, measuring EPSC or IPSC amplitudes in the dorsal striatum upon increasing stimulation current intensities. Corticostriatal glutamatergic synapses of Glu-GAP43^−/−^ mice showed a higher excitability than those of control mice (Fig. 6*A*; GAP43^fl/fl^, *n* = 6 cells, Glu-GAP43^−/−^, *n* = 7 cells, *t*_(11) _= 3.142, Glu-GAP43^−/−^ vs GAP43^fl/fl^ mice, *p* = 0.0094 at 50 µA, recorded at −70 mV), without any overt alterations found in GABAergic transmission (Fig. 6*B*; *t*_(11) _= 0.8499, Glu-GAP43^−/−^ vs GAP43^fl/fl^ mice, *p* = 0.4135 at 50 µA, recorded by clamping the potential at 0 mV, which is close to the reversal potential of glutamatergic receptors, in order to minimize the influence of AMPA and NMDA receptor currents and allowing Cl^−^ flow through GABA_A_ receptors). The increase of release probability of glutamatergic inputs in Glu-GAP43^−/−^ mice was further supported by a significant decrease in the basal PPR of these synapses compared with those in GAP43^fl/fl^ littermates (Fig. 6*C*; GAP43^fl/fl^, *n* = 6 cells, Glu-GAP43^−/−^, *n* = 6 cells, *U* = 3.00, Glu-GAP43^−/−^ vs GAP43^fl/fl^ mice, *p* = 0.0152 at 75 ms interstimulus interval recorded at −70 mV). Again, GABAergic inputs of Glu-GAP43^−/−^ mice did not show any significant change (Fig. 6*D*; GAP43^fl/fl^, *n* = 5 cells, Glu-GAP43^−/−^, *n* = 7 cells, *U* = 9.00, Glu-GAP43^−/−^ vs GAP43^fl/fl^, mice *p* = 0.2020 at 200 ms interstimulus interval recorded at 0 mV).

To assess whether the increased excitability of glutamatergic synapses of Glu-GAP43^−/−^ mice is due to an increase in the release probability or in the size of the pool of synaptic vesicles prepared to release neurotransmitters in response to action potentials, the RRP size was calculated from the cumulative amplitude plots as the *y*-intercept from a linear fit of the steady-state values attained during high-frequency trains (100 stimuli at 40 Hz; Fig. 6*E*,*F*). Under these conditions, glutamatergic synapses of Glu-GAP43^−/−^ mice (recording EPSCs at −70 mV) showed an increased RRP size compared with GAP43^fl/fl^ mice (Fig. 6*G*, left; GAP43^fl/fl^, *n* = 6 cells, Glu-GAP43^−/−^, *n* = 6 cells, *t*_(10) _= 2.623, Glu-GAP43^−/−^ vs GAP43^fl/fl^ mice, *p* = 0.0255). This experimental approach also allows measuring the replenishment rate of the RRP by analyzing the slope of the steady-state cumulative amplitude plots. No differences between glutamatergic inputs of Glu-GAP43^−/−^ and control mice was found (Fig. 6*G*, middle; GAP43^fl/fl^, *n* = 6 cells, Glu-GAP43^−/−^, *n* = 6 cells, *U* = 16, Glu-GAP43^−/−^ vs GAP43^fl/fl^ mice, *p* = 0.8182). In addition, estimation of the Pves values did not reveal any change between Glu-GAP43^−/−^ and control mice (Fig. 6*G*, right; GAP43^fl/fl^, *n* = 6 cells, Glu-GAP43^−/−^, *n* = 6 cells, *t*_(10) _= 0.6382, Glu-GAP43^−/−^ vs GAP43^fl/fl^ mice, *p* = 0.5377), thus corroborating that the increased excitability of glutamatergic synapses is not due to an enhanced release probability but to an augmented RRP size. In line with other aforementioned data, GABAergic inputs of Glu-GAP43^−/−^ mice did not show any significative change in RRP size (Fig. 6*H*, left; GAP43^fl/fl^, *n* = 6 cells, Glu-GAP43^−/−^, *n* = 6 cells; *t*_(10) _= 0.2231, Glu-GAP43^−/−^ vs GAP43^fl/fl^ mice, *p* = 0.8280), RRP replenishment rate (Fig. 6*H*, middle; GAP43^fl/fl^, *n* = 6 cells, Glu-GAP43^−/−^, *n* = 6 cells; *t*_(10) _= 0.5244, Glu-GAP43^−/−^ vs GAP43^fl/fl^ mice, *p* = 0.6114) or Pves (Fig. 6*H*, right; GAP43^fl/fl^, *n* = 6 cells, Glu-GAP43^−/−^, *n* = 6 cells; *t*_(10) _= 0.8428, Glu-GAP43^−/−^ vs GAP43^fl/fl^ mice, *p* = 0.4190).

Taken together, these electrophysiological data align with the behavioral and anatomical results shown above and support an increased glutamate release from corticostriatal terminals upon GAP43 deletion, which may underlie an enhanced synaptic excitability and the abrogation of endocannabinoid/CB_1_R-mediated LTD.

### Chemogenetic inhibition of corticostriatal afferences reverts the hyperactive phenotype of Glu-GAP43^−/−^ mice

**Figure 7.**
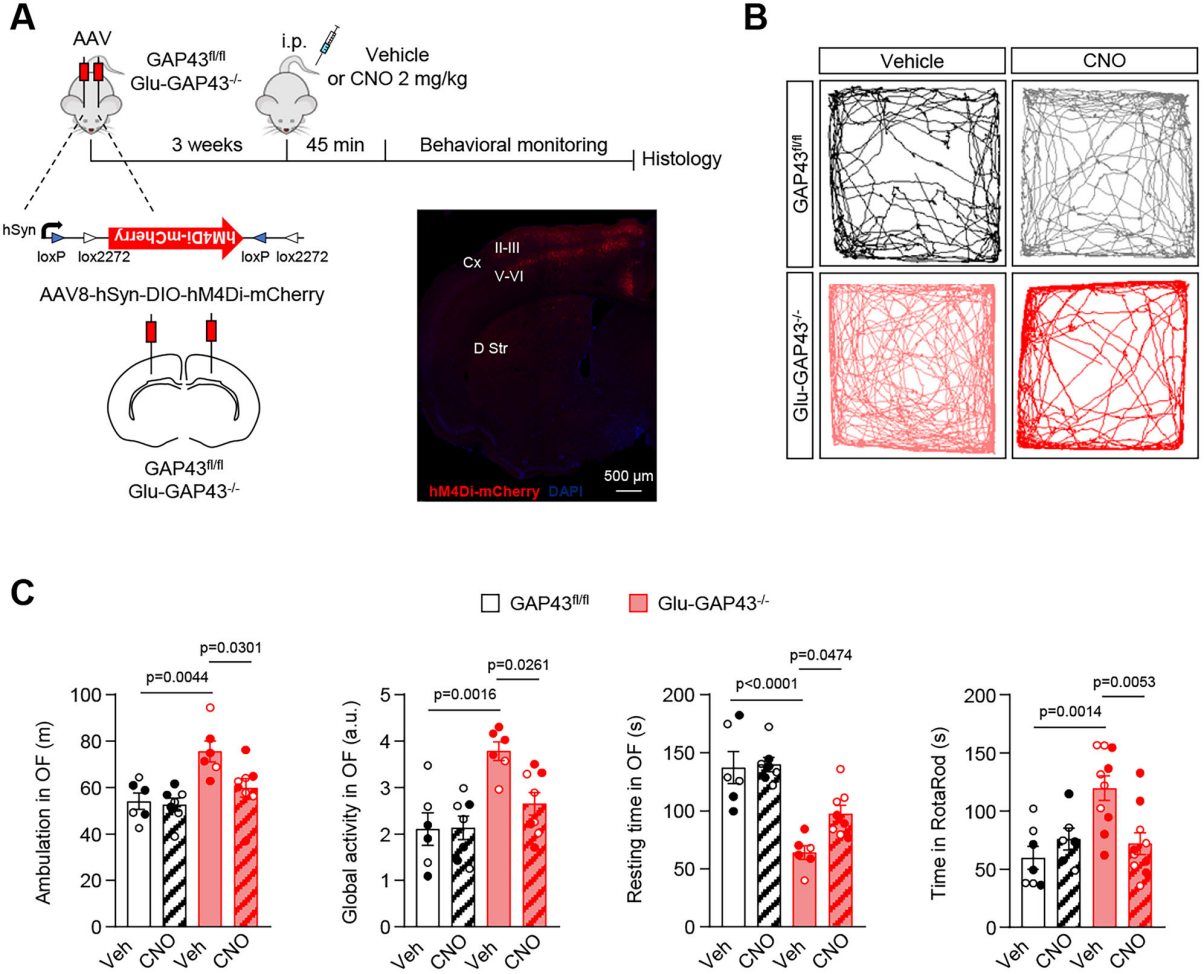
Chemogenetic inhibition of corticostriatal afferences reverts the hyperactive phenotype of Glu-GAP43^−/−^ mice. ***A***, Timeline of the experiments. Glu-GAP43^−/−^ or GAP43^fl/fl^ mice were injected stereotaxically in the frontal cortex with AAV8-hSyn-DIO-hM4Di-mCherry (schematic vector is depicted). Three weeks after, animals were injected intraperitoneally with saline vehicle or CNO (2 mg/kg), and 45 min later motor behavior was analyzed. A representative image of an injected brain hemisphere is shown. Cx, cortex; D Str, dorsal striatum; II–III and V–VI, cortical layers II–III and V–VI. ***B***, Representative trajectory maps in a 10 min open-field test of Glu-GAP43^−/−^ and GAP43^fl/fl^ mice treated with saline vehicle or CNO. ***C***, Ambulation (total distance traveled, m), global activity (a.u.), and resting time (s) in a 10 min open-field (OF) test, as well as RotaRod performance (time to fall, s), in Glu-GAP43^−/−^ mice and their GAP43^fl/fl^ littermates (means ± SEM; *n* = 6–10 mice per group; males, empty circles; females, filled circles; *p* values obtained by two-way ANOVA with Tukey's multiple-comparisons test).

To further support the involvement of an overactivation of GAP43-deficient corticostriatal projections in novelty-induced hyperactivity, we selectively manipulated excitatory transmission in vivo by the “designer receptor exclusively activated by designer drug” (DREADD) chemogenetic technique (Armbruster et al., 2007). For this purpose, we stereotaxically injected GAP43^fl/fl^ and Glu-GAP43^−/−^ mice with a recombinant adeno-associated viral vector encoding an engineered G_i_-protein-coupled DREADD fused to mCherry (AAV8-hSyn-DIO-hM4Di-mCherry). Injections were performed bilaterally into the frontal (including motor) cortex, where the somata of the glutamatergic afferents projecting onto the dorsal striatum reside. The expression of the hM4Di transgene was dependent on Cre recombinase and was driven by the neuronal human α-synapsin-1 (hSyn) promoter. Three weeks after vector injection, the somatodendritic expression of the DREADD-mCherry protein was evident (Fig. 7*A*). Then, animals were acutely injected intraperitoneally with either the hM4Di-selective agonist CNO (at 2 mg/kg body weight) or saline vehicle as control, and behavioral tests were conducted 45 min later. DREADD-expressing Glu-GAP43^−/−^ mice treated with vehicle, in which the DREADD conceivably remains inactive (Roth, 2016), reproduced the aforementioned hyperactive phenotype of naive Glu-GAP43^−/−^ animals in the open-field test (Fig. 7*B*,*C*; GAP43^fl/fl^ saline, *n* = 6 mice, GAP43^fl/fl^ CNO, *n* = 7 mice, Glu-GAP43^−/−^ saline, *n* = 6 mice, Glu-GAP43^−/−^ CNO, *n* = 8 mice; ambulation: *F*_(3,23) _= 7.236; Glu-GAP43^−/−^ saline vs GAP43^fl/fl^ saline, *p* = 0.0044; global activity: *F*_(3,23) _= 8.053; Glu-GAP43^−/−^ saline vs GAP43^fl/fl^ saline, *p* = 0.0016; resting time: *F*_(3,22) _= 16.80; Glu-GAP43^−/−^ saline vs GAP43^fl/fl^ saline, *p* < 0.0001) as well as in the RotaRod test (Fig. 7*C*; GAP43^fl/fl^ saline, *n* = 7 mice, GAP43^fl/fl^ CNO, *n* = 6 mice, Glu-GAP43^−/−^ saline, *n* = 10 mice, Glu-GAP43^−/−^ CNO, *n* = 10 mice; *F*_(3,29) _= 7.307; Glu-GAP43^−/−^ saline vs GAP43^fl/fl^ saline, *p* = 0.0014). CNO had no effect on GAP43^fl/fl^ mice (Fig. 7*B*,*C*; ambulation: GAP43^fl/fl^ CNO vs GAP43^fl/fl^ saline, *p* = 0.9932; global activity: GAP43^fl/fl^ CNO vs GAP43^fl/fl^ saline, *p* = 0.9998; resting time: GAP43^fl/fl^ CNO vs GAP43^fl/fl^ saline, *p* = 0.9961; RotaRod: GAP43^fl/fl^ CNO vs GAP43^fl/fl^ saline, *p* = 0.7560). In contrast, the CNO/DREADD-induced inhibition of Cre-expressing cortical afferences decreased the hyperlocomotor phenotype of Glu-GAP43^−/−^ mice down to the baseline levels of GAP43^fl/fl^ littermates (Fig. 7*C*; ambulation: Glu-GAP43^−/−^ CNO vs Glu-GAP43^−/−^ saline, *p* = 0.0301; global activity: Glu-GAP43^−/−^ CNO vs Glu-GAP43^−/−^ saline, *p* = 0.0261; resting time: Glu-GAP43^−/−^ CNO vs Glu-GAP43^−/−^ saline, *p* = 0.0474; RotaRod: Glu-GAP43^−/−^ CNO vs Glu-GAP43^−/−^ saline, *p* = 0.0053). These data, by showing that silencing the corticostriatal circuit of Glu-GAP43^−/−^ mice is sufficient to regain a wild-type–like motor phenotype, further support the corticostriatal substrate of the hyperactivity observed in these animals.

## Discussion

Here, we show that the selective deletion of GAP43 from dorsal telencephalic glutamatergic neurons causes novelty-induced hyperactivity, plausibly owing to an overactivity of corticostriatal synaptic transmission and plasticity that, in turn, leads to an excessive activation of dorsal-striatum MSNs upon exposure of the animal to an unfamiliar environment. To date, the role of GAP43 in the control of motor behavior had been scarcely studied. In line with our results, a previous report showed a hyperactive phenotype of GAP43^−/−^ postnatal mice (Metz and Schwab, 2004). Restraining novelty-induced hyperactivity is, to our knowledge, the first specific function that has ever been ascribed to GAP43 in the corticostriatal circuitry.

The striatum is a key hub in the basal ganglia for integrating neural information from various cortical regions in concert with different downstream subcortical structures. These diverse cortical inputs allow the basal ganglia circuitry to regulate many physiological processes, ranging from a basic level of motor output to a higher level of cognitive and affective functions (Kuo and Liu, 2019). Corticostriatal projections are topographically well organized within the striatum in dorsoventral and mediolateral gradients that delineate motor, cognitive, and affective functions. Thus, major motor and somatosensory cortical outputs extend to the central and lateral parts of the dorsal striatum, which is largely involved in motor sequencing and habit-related and stimulus response-driven functions, while prominently associative frontal cortical areas project onto the central to medial dorsal striatum, which is mostly involved in goal-directed motor learning. Afferences from the allocortex project onto the reward-driving ventral/limbic striatum (Kuo and Liu, 2019). Our data reveal that, upon exposure of Glu-GAP43^−/−^ mice to a novel environment, a selective increase of c-Fos immunolabeling occurs in the prefrontal cortex, dorsal striatum, nucleus accumbens, and hippocampal formation but not in the amygdala, which might point to a risk-taking behavioral trend in those animals. However, when comparing with GAP43^fl/fl^ animals, this increase in c-Fos expression was only statistically significant in the dorsal striatum, thus supporting a stronger contribution of GAP43 to the control of dorsal-striatum over hippocampal/limbic-dependent functions, at least in this experimental setting. Cortical projections to the dorsal and ventral striatum are anatomically and biochemically distinct (Basile et al., 2021). We are aware that here we have not explored the precise pattern of GAP43 expression across different cortical subregions, but our data strongly support a selective relevance of the pool of GAP43 molecules located on cortical neurons projecting onto the dorsal striatum rather than the ventral striatum. We have neither delimitated precise subregions along the mediolateral axis of the dorsal striatum, but our data point to a more prominent dorsocentral-to-dorsolateral contribution to the observed effects as Glu-GAP43^−/−^ mice seem to fail in their ability to habituate to the environment, showing an enhanced neuronal activity in response to novel but behaviorally irrelevant stimuli.

GAP43 coordinates the release and recycling of neurotransmitter vesicles, and this constitutes a plausible mechanism by which it controls synaptic potentiation (Holahan, 2017). Thus, phosphorylated GAP43 interacts at the presynaptic terminal with vesicle-associated proteins such as SNAP25, VAMP (Haruta et al., 1997), and rabaptin-5, which increases endocytosis and recycling of vesicles in vitro (Neve et al., 1998). However, our ex vivo electrophysiological results show that GAP43 deletion in corticostriatal glutamatergic terminals increases the size of the pool of synaptic vesicles that are prepared to release glutamate, with no differences in the vesicular replenishment/recycling rate. This alteration might be due to a circuit-based specificity of the GAP43 pool located on corticostriatal terminals and/or to a functional response that relies on its phosphorylation–dephosphorylation cycle. An absence of GAP43 expression might also bring some compensational changes that dysregulate the machinery of neurotransmitter release, thus leading to an accumulation of vesicles at the presynaptic terminal. Changes in the synaptic efficacy of cortical inputs to striatal MSNs can contribute to motor learning (Pisani et al., 2005). In the present study, we unveil an abrogation of mGluRI-dependent corticostriatal LTD in concert with overt motor alterations in Glu-GAP43^−/−^ mice. This type of LTD is known to be mediated, at least in part, by postsynaptic endocannabinoid release and presynaptic CB_1_R activation, which reduces glutamate output from cortical afferences (Gerdeman et al., 2002). In line with these electrophysiology data, pharmacological activation of (conceivably G_i_-protein-coupled) CB_1_Rs as well as G_i_-protein-evoked chemogenetic silencing of corticostriatal afferences rescued the hyperactive phenotype of Glu-GAP43^−/−^ mice, therefore suggesting that, upon GAP43 deletion from excitatory terminals, CB_1_Rs may not be sufficiently active to suppress the abnormally high number of glutamate vesicles ready to be released. We have recently described an inhibitory interaction between GAP43 and CB_1_R in mossy cells of the hippocampal dentate gyrus (Maroto et al., 2023). Hence, the loss of corticostriatal LTD and the associated increase in glutamatergic transmission upon GAP43 deletion that we report here may not be explained by this type of inhibitory protein–protein interaction but by a GAP43-mediated maintenance of tonic CB_1_R receptor function, thus pointing to a fine synapse-type specificity of the GAP43/CB_1_R cross talk that might be different in the striatum and the dentate gyrus of the hippocampus.

It is worth noting that, although we found GAP43 mRNA and protein expression in MSNs of the dorsal striatum, and these cells are long known to express as well large amounts of CB_1_Rs (Lovinger, 2010; see also Extended Data Fig. 5-1*B*), to date we have failed to detect any overt behavioral alteration in our GABA-GAP43^−/−^ mouse line (present study; Maroto et al., 2023). In the future, an additional series of tests could be performed on these animals, including drug-seeking/addictive behaviors, compulsive-like behaviors, catalepsy, and amphetamine-induced sensitization, aimed to unveil other potential phenotypic alterations. It is uncertain whether disarrangements on the presynaptic terminal of MSNs upon GAP43 deletion can occur, as the functional importance of GAP43 at the inhibitory synaptic machinery seems to be weaker than at excitatory hippocampal (Maroto et al., 2023) and corticostriatal terminals (present study). In this respect, it could be interesting to conduct electrophysiology experiments on MSN-MSN synapses of GABA-GAP43^−/−^ and GAP43^fl/fl^ mice to evaluate the actual relevance of GAP43 in striatal inhibitory transmission.

Studies conducted in patients and animal models support that alterations in corticostriatal circuitry are involved in various neuropsychiatric disorders, including ADHD (Kuo and Liu, 2019). In addition, reduced GAP43 levels were found in the frontal cortex and hippocampus of an ADHD rat model (Almeida et al., 2023). Several animal models of those diseases that show hyperactive phenotypes also display alterations in the expression of glutamatergic transmission elements such as the AMPA receptor GluA1 subunit (Wiedholz et al., 2008; Aitta-Aho et al., 2019) and the NMDA receptor GluN2A subunit (Farsi et al., 2023). Neurological and psychiatric disorders inflict significant health and socioeconomic burden to our society. The development of new neuropsychiatric drugs, notably for conditions like ADHD, schizophrenia, and bipolar disorder, is a current clinical challenge. Unfortunately, animal models cannot fully replicate the wide array of symptoms found in those so heterogeneous and multifactorial brain diseases. Thus, the Glu-GAP43^−/−^ mouse could become a novel and robust model to study the corticostriatal component of novelty-induced hyperactivity, and the subsequent habituation to the familiar environment, as a phenotypic trait of ADHD, schizophrenia, or the maniac phase of bipolar disorder.
