# Supplementary figures and images for: GAP43 Located on Corticostriatal Terminals Restrains Novelty-Induced Hyperactivity in Mice
Source: J Neurosci. 2024 Aug 21;44(39):e0701242024. doi: 10.1523/JNEUROSCI.0701-24.2024 (PMC11426381; doi:10.1523/JNEUROSCI.0701-24.2024)

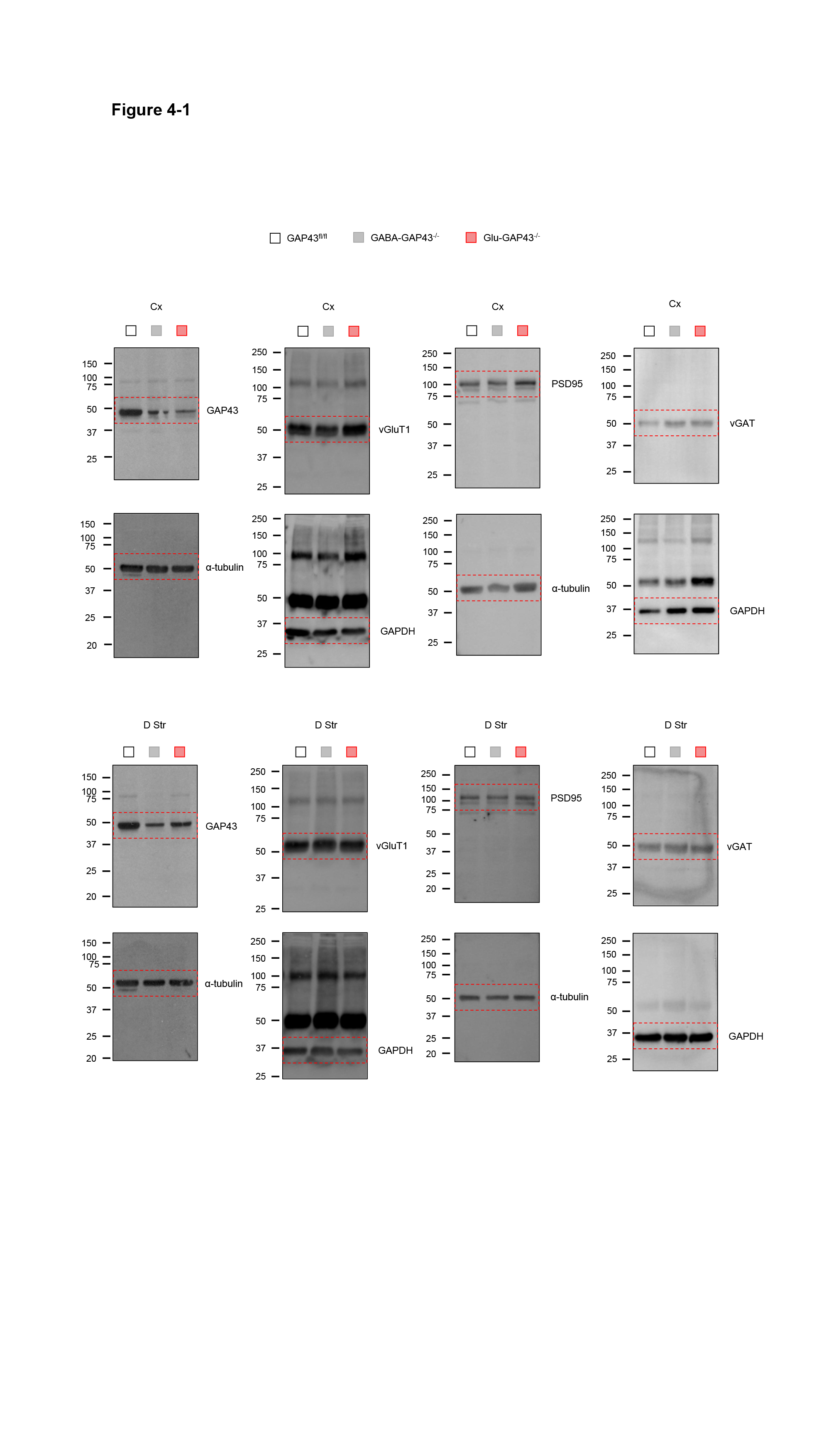

Supplement: Fig 4-1 — Uncropped version of the blots shown in Fig 4. Download Fig 4-1, TIF file. [file jneuro-44-e0701242024-s001.tif]

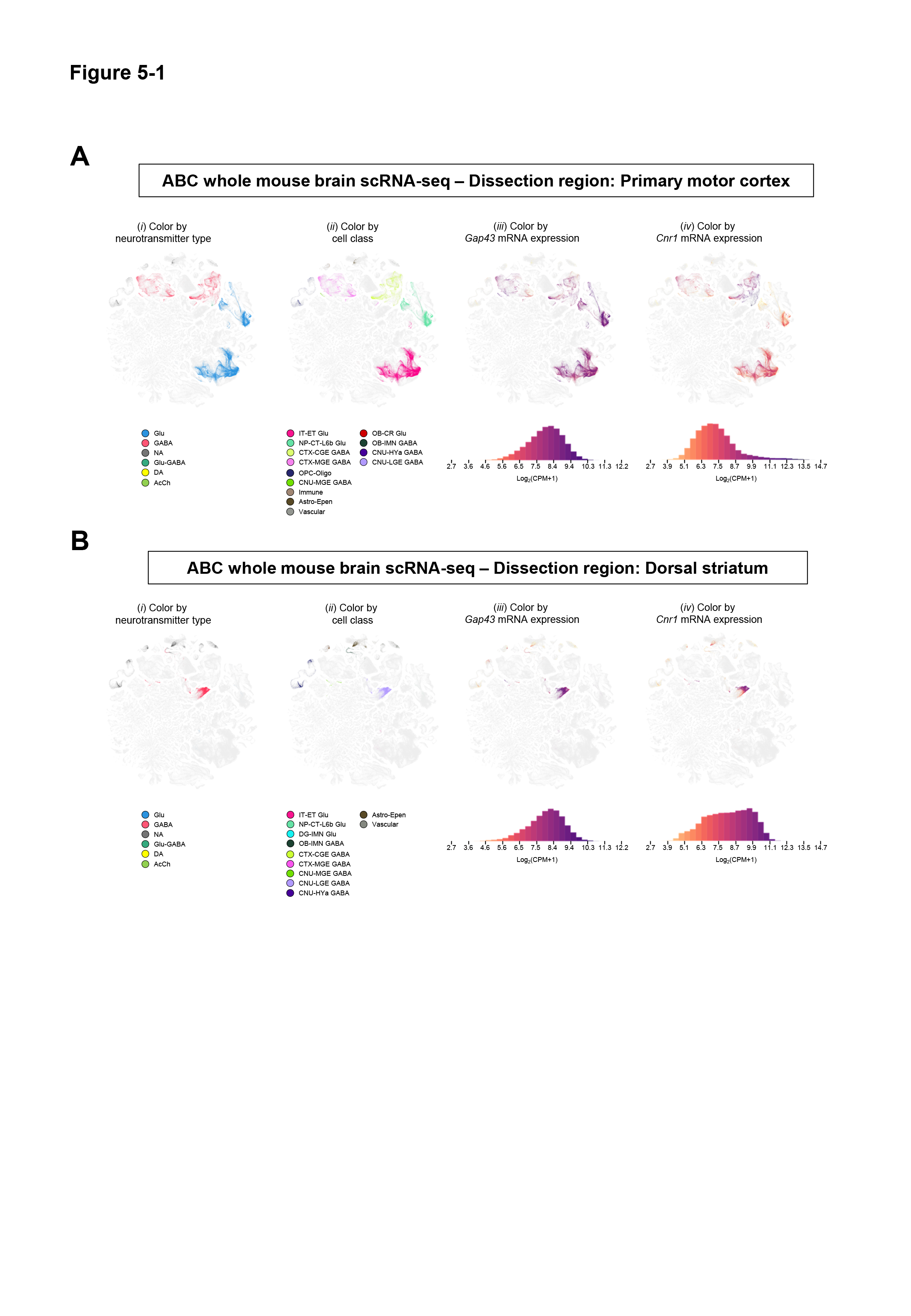

Supplement: Fig 5-1 — Single-cell transcriptomic analysis of Gap43 and Cnr1 mRNAs in mouse primary motor cortex and dorsal striatum. The single-cell RNA sequencing data from the Whole Mouse Brain Transcriptomic Cell Type Atlas was accessed using the online tool provided by the Allen Brain Map initiative (https://knowledge.brain-map.org/abcatlas, accessed on 12/06/2024). The dataset was filtered by dissection region [either primary motor cortex (MOp) or dorsal striatum (STRd)] prior to plotting the corresponding Uniform Manifold Approximation and Projection (UMAP) maps. A. UMAP representation of MOp cells (∼248,000 individual cells) colored by (i) neurotransmitter type, (ii) cell class (doi: 10.1038/s41586-023-06812-z), (iii) Gap43 mRNA expression (in counts per million bases, CPM), and (iv) Cnr1 mRNA expression (in CPM). As inferred from the overlay of the UMAPs, coincidental neuronal classes co-expressing Gap43 and Cnr1 mRNAs include projection neurons (IT-ET Glu). B. UMAP representation of dorsal STRd cells (∼55,600 individual cells) colored by (i) neurotransmitter type, (ii) cell class (doi: 10.1038/s41586-023-06812-z), (iii) Gap43 mRNA expression (in CPM), and (iv) Cnr1 mRNA expression (in CPM). As inferred from the overlay of the UMAPs, coincidental neuronal classes co-expressing Gap43 and Cnr1 mRNAs include medium spiny neurons (CNU-LGE GABA). Neurotransmitter type: AcCh, cholinergic; DA, dopaminergic; GABA, GABAergic; Glu, glutamatergic; NA, noradrenergic. Cell class: Astro, astrocyte; CGE, caudal ganglionic eminence; CNU, cerebral nuclei; CR, Cajal–Retzius; CT, corticothalamic; CTX, cerebral cortex; Epen, ependymal; ET, extratelencephalic; HYa, anterior hypothalamic; IMN, immature neuron; IT, intratelencephalic; L6b, layer 6b; LGE, lateral ganglionic eminence; MGE, medial ganglionic eminence; NP, near-projecting; OB, olfactory bulb; Oligo, oligodendrocyte; OPC, oligodendrocyte precursor cell. Download Fig 5-1, TIF file. [file jneuro-44-e0701242024-s002.tif]
